# Neuroepigenetic signatures of age and sex in the living human brain

**DOI:** 10.1038/s41467-019-11031-0

**Published:** 2019-07-03

**Authors:** Tonya M. Gilbert, Nicole R. Zürcher, Mary C. Catanese, Chieh-En J. Tseng, Maria A. Di Biase, Amanda E. Lyall, Baileigh G. Hightower, Anjali J. Parmar, Anisha Bhanot, Christine J. Wu, Matthew L. Hibert, Minhae Kim, Umar Mahmood, Steven M. Stufflebeam, Frederick A. Schroeder, Changning Wang, Joshua L. Roffman, Daphne J. Holt, Douglas N. Greve, Ofer Pasternak, Marek Kubicki, Hsiao-Ying Wey, Jacob M. Hooker

**Affiliations:** 1 Athinoula A. Martinos Center for Biomedical Imaging, Department of Radiology, Massachusetts General Hospital, Harvard Medical School, Charlestown, MA 02129 USA; 2 Psychiatry Neuroimaging Laboratory, Departments of Psychiatry and Radiology, Brigham and Women’s Hospital, Harvard Medical School, Boston, MA 02215 USA; 3 Department of Psychiatry, Massachusetts General Hospital, Harvard Medical School, Boston, MA 02114 USA

**Keywords:** Positron-emission tomography, Epigenetics in the nervous system

## Abstract

Age- and sex-related alterations in gene transcription have been demonstrated, however the underlying mechanisms are unresolved. Neuroepigenetic pathways regulate gene transcription in the brain. Here, we measure in vivo expression of the epigenetic enzymes, histone deacetylases (HDACs), across healthy human aging and between sexes using [^11^C]Martinostat positron emission tomography (PET) neuroimaging (*n* = 41). Relative HDAC expression increases with age in cerebral white matter, and correlates with age-associated disruptions in white matter microstructure. A post mortem study confirmed that HDAC1 and HDAC2 paralogs are elevated in white matter tissue from elderly donors. There are also sex-specific in vivo HDAC expression differences in brain regions associated with emotion and memory, including the amygdala and hippocampus. Hippocampus and white matter HDAC expression negatively correlates with emotion regulation skills (*n* = 23). Age and sex are associated with HDAC expression in vivo, which could drive age- and sex-related transcriptional changes and impact human behavior.

## Abstract (web summary)

Gene transcription is known to vary with age and sex, although the underlying mechanisms are unresolved. Here, the authors show that epigenetic enzymes known as HDACs, which regulate gene transcription, are increasingly expressed with age in the living human brain, with sex differences also observed.

## Introduction

Age and sex represent two of the major biological variables that influence human health and disease. Aging is the main risk factor for neurodegenerative disease^1,2^, and is associated with progressive deterioration of brain structure and function^3^. Sex differences impact the incidence, severity, and treatment response in neurological and psychiatric disorders^4,5^. While age- and sex-related alterations in gene transcription have been demonstrated^6–9^, the underlying molecular mechanisms are unresolved. Genetic background alone cannot fully explain the relationship between these variables and changes in gene transcription. Epigenetic mechanisms, which integrate environmental and genetic factors, have a profound impact upon gene regulation that when altered, may drive disease risk^10,11^.

Dysregulation of histone deacetylases (HDACs), a family of epigenetic enzymes, could tip the balance from healthy to pathological aging. Hypoacetylation of specific histone marks is linked to age-related neurodegenerative diseases, as demonstrated by preclinical and post mortem studies^12,13^. In rodent models, HDAC expression^14,15^ and enzymatic activity^16^ increase with age in the hippocampus, leading to reductions in processes including histone acetylation^17–19^, transcription of synaptic plasticity genes^19–21^, and memory formation^19,21^. HDAC inhibition alleviates age-related phenotypes^12,17–19^, such as deficiencies in gene transcription and cognitive performance that are present in human aging^6,22^. In the young adult human brain, HDAC expression is lowest in the white matter^23^. The effects of altered HDACs across lifespan are not fully understood. Interestingly, the efficiency of oligodendrocyte-mediated remyelination decreases with age in rodents, due to altered recruitment of HDACs at the promoter regions of myelin-associated genes^24^. Furthermore, HDAC inhibition reduces excitotoxicity^25^ and is protective against white matter injury in rodent disease models^26,27^.

Sexually dimorphic regulation of gene transcription by HDACs may contribute to differences in the prevalence of neurological and neuropsychiatric disorders between females and males^4^. For example, sex-specific HDAC expression patterns could impart susceptibility to memory-related disorders, supported by evidence from rodents of transient hypoacetylation in the hippocampus of females compared to males during early development^28^ and the positive relationship between histone acetylation and estrogen-induced memory enhancement in females^29^. Further, HDACs modulate and respond to sex steroid hormones^29^, influence sexual behavior^30^, and alter socio-emotional circuits in the limbic system^31^. Moreover, HDACs play a fundamental role in male sex determination^32^, female X-chromosome inactivation^33^, and genomic imprinting^34^.

Although HDACs are implicated in age- and sex-related vulnerability to disease through preclinical and post mortem studies, differences exist between these models and living humans at both molecular and systems levels. In humans, non-invasive measurement of in vivo HDAC expression is essential to investigate the contribution of neuroepigenetic mechanisms to age- and sex-related alterations in gene transcription. Using [^11^C]Martinostat^23,35–38^, an HDAC positron emission tomography (PET) radiotracer, we previously mapped HDAC expression in healthy young-adults^23^. Here, we measure relative [^11^C]Martinostat uptake in the brains of healthy individuals across the adult lifespan, and compare HDAC expression patterns between females and males. We then apply simultaneously acquired diffusion magnetic resonance (MR) imaging and cognitive performance testing to relate HDAC expression levels with alterations in white matter microstructure and human socio-emotional behavior, respectively. We find that relative HDAC expression increases with age in cerebral white matter and correlates with alterations in white matter microstructure. We also identify sex-specific HDAC expression differences in brain regions associated with socio-emotional processes, including the amygdala and hippocampus. We provide preliminary evidence that HDAC expression in both the hippocampus and white matter negatively correlates with emotion regulation skills. We propose that HDACs are strongly associated with the major biological variables of age and sex, and may represent potential targets to modify age- and sex-related pathophysiology.

## Results

### Study design

To characterize HDAC expression patterns across the adult lifespan and between females and males, healthy adults (*n* = 41 subjects, mean age ± standard deviation = 41 ± 17 years, 18–79-years-old, 20 females, 21 males) underwent simultaneous MR-PET with [^11^C]Martinostat, a radiotracer selective for HDAC paralogs 1, 2, 3, and putatively 6^23,35–38^. [^11^C]Martinostat was previously found to engage HDAC1, HDAC2, and HDAC3 in both in vitro assays using recombinant proteins^35^ and ex vivo assays using human brain tissue^23^, while HDAC6 was engaged only in in vitro assays^23,35^.

### [^11^C]Martinostat SUVR is a surrogate measure for *V*_T_

Given the risks and invasiveness of arterial blood sampling, we first determined that standard uptake value (SUV) normalized to whole brain mean (standard uptake value ratio, SUVR) from 60 to 90 min post radiotracer injection is an appropriate surrogate measure for the distribution volume (*V*_T_) in a subset of healthy young and old adults. Regional SUVR were strongly correlated with *V*_T_ values derived from a two-tissue compartmental model (2TCM), using metabolite-corrected arterial plasma as an input function (all Pearson’s *r* ≥ 0.90, all *P* < 0.0001, Supplementary Fig. 1). SUVR was therefore used as the primary outcome measure to determine relative changes in [^11^C]Martinostat uptake^38–40^.

### SUVR increases with age in the white matter

**Fig. 1:**
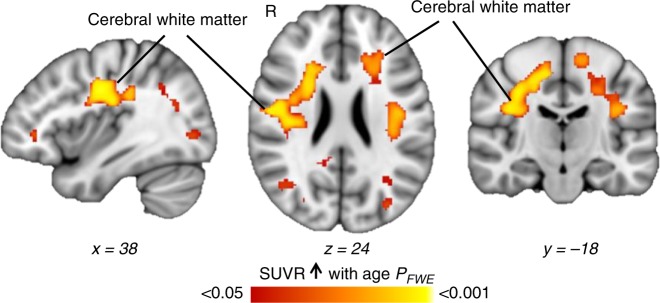
[^11^C]Martinostat uptake increases with age in the white matter. Voxel-wise correlations of SUVR with age, controlled for sex, total grey matter volume/intracranial volume, white matter volume/intracranial volume, and cerebrospinal fluid volume/intracranial volume (*n* = 41). Statistical maps were overlaid onto the MNI 1 mm template in radiological orientation (family wise error rate (FWE) corrected for multiple comparisons, *P*_*FWE*_ < 0.05; non-parametric permutation testing *n* = 10,000 permutations). Red–yellow represents regions significantly increased with age

First, voxel-wise analysis correlating [^11^C]Martinostat SUVR with age was performed while controlling for sex and brain volumes^41^ (grey matter, white matter, and cerebrospinal fluid volumes normalized by estimated total intracranial volume) (*P*_*FWE*_ < 0.05, Fig. 1, Supplementary Note 1, Supplementary Fig. 2). We observed a significant increase in HDAC expression with age in the cerebral white matter, but not the corpus callosum (Fig. 1). Analysis in native space (i.e. individual anatomically defined regions for every subject) supported these results (Supplementary Fig. 3a). The native space cerebral white matter masks were eroded in order to assess SUVR changes in deep white matter, where physical contact with the ventricles (a major source of partial volume effects) was limited. SUVR remained strongly correlated with age in the eroded masks (Supplementary Fig. 3b). Partial volume correction was applied using two complementary approaches: (1) voxel-wise correlations of SUVR with age were restricted to white matter, controlled for sex and region-based voxel-wise (RBV) correction was applied (Supplementary Fig. 4a) and (2) SUVR was extracted from cerebral white matter in native space using the geometric transfer matrix (GTM) and correlated to age (Supplementary Fig. 4b). Partial volume corrected SUVR and age were correlated in the cerebral white matter, confirming our findings. Further, these results were not driven by body mass index (BMI) and were recapitulated using a secondary normalization method (Supplementary Notes 2 and 3, Supplementary Figs. 5–7). Interestingly, SUVR in the cerebral white matter increased with age only in mid to late adulthood, as demonstrated by correlation analysis split at the median age of 35 years. A positive correlation was found in subjects older than 35 years (Spearman’s *r* = 0.44, *P* < 0.05), whereas no correlation was observed in subjects equal or younger than 35 years (Spearman’s *r* = −0.082, *P* = 0.73, Supplementary Fig. 8).

Second, based on previous ex vivo studies that reported elevated HDAC expression and deficient histone acetylation in the hippocampus of aged rodents^14,17–19,42^, the hippocampus was selected for a priori region of interest (ROI) analysis and GTM partial volume correction was applied. However, contrary to rodent studies, HDAC expression and age were not strongly correlated in the human hippocampus, despite the presence of a trend (Spearman’s *r* = 0.29, *P* = 0.062, Supplementary Fig. 9).

### SUVR correlates with alterations in white matter structure

**Fig. 2:**
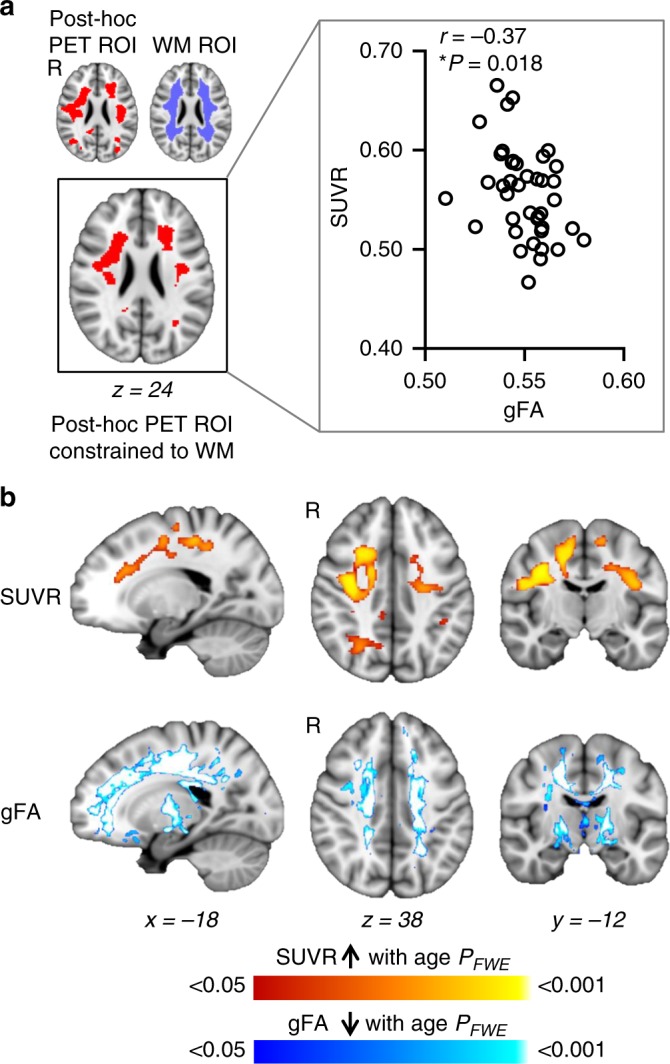
[^11^C]Martinostat uptake correlates and co-localizes with alterations in white matter microstructure. **a** (*Left*) A post-hoc PET ROI was created from a binary mask of the entire statistical map in Fig. 1 (red, depicting regions where SUVR increased with age), and was constrained to white matter. ROIs are overlaid onto the MNI 1 mm template in radiological orientation. (*Right*) Spearman correlation analysis between SUVR and gFA values extracted from the post-hoc PET ROI constrained to white matter (*n* = 40). **b** Voxel-wise correlations of (*Top*) SUVR increases with age, controlled for sex, total grey matter volume/intracranial volume, white matter volume/intracranial volume, and cerebrospinal fluid volume/intracranial volume (*n* = 41) and (*Bottom*) gFA decreases with age, controlled for sex (*n* = 40). Statistical maps were overlaid onto the MNI 1 mm template in radiological orientation (*P*_*FWE*_ < 0.05; non-parametric permutation testing *n* = 10,000 permutations). Red–yellow represents regions where SUVR significantly increased with age and blue-light blue represents regions where gFA significantly decreased with age

Taking advantage of simultaneous MR-PET methodology, we sought to determine whether age-associated increases in HDAC expression are linked with changes in white matter microstructure. We analyzed generalized fractional anisotropy (gFA), a diffusion metric similar to fractional anisotropy (FA), which represents white matter microstructural organization. Analysis of gFA is believed to be less sensitive to measurement errors that result from crossing fibers compared to FA^43–45^. First, ROI analysis guided by our PET finding (Fig. 1) was performed to determine the relationship between [^11^C]Martinostat SUVR and gFA. SUVR and gFA values were extracted from the post-hoc PET ROI where SUVR increased with age, restricted to white matter areas. See Fig. 2a for ROI description. In this ROI, SUVR negatively correlated with gFA (Spearman’s *r* = −0.37, *P* = 0.018, Fig. 2a). Next, voxel-wise analysis correlating gFA with age while controlling for sex (*P*_*FWE*_ < 0.05, Fig. 2b) was performed across the whole brain to determine co-localization between alterations in white matter microstructure and increases in HDAC expression. Age-related gFA decreases showed extensive spatial overlap with age-related SUVR increases (Fig. 2b).

### HDAC1 and HDAC2 increases in elderly white matter tissue

**Fig. 3:**
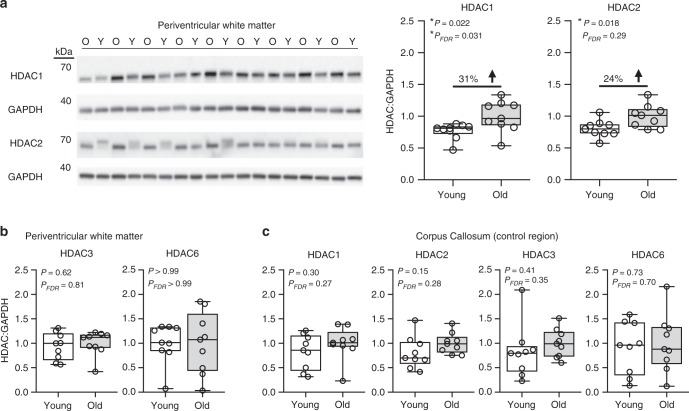
HDAC1 and HDAC2 expression is increased in the white matter of elderly donors. Whole-cell lysates were prepared from post mortem periventricular white matter and corpus callosum tissues. Protein expression of HDAC paralogs (1, 2, 3, and 6) were compared between younger donors (*n* = 9, 16–21 years -old) and older donors (*n* = 9, 76–100-years-old) by western blotting (two-tailed unpaired *t*-test; false discovery rate (FDR) corrected for multiple comparisons). For each paralog, immunoreactive band intensity was normalized by GAPDH and scaled to a mean of 1. Box plots display median, first quartile, third quartile, and range (min–max) from **a**, **b** periventricular white matter and **c** corpus callosum. See Supplementary Figs. 10 and 11 for western blot images

To investigate age-related increases of [^11^C]Martinostat uptake at the molecular level, post mortem human brain tissue was obtained from older donors (*n* = 9, mean age ± standard deviation = 85 ± 8 years) and younger donors (*n* = 9, mean age = 18 ± 1 year) matched for sex (*n* = 4 females and *n* = 5 males in each group). Periventricular white matter (PVM) was chosen because [^11^C]Martinostat SUVR increased with age in PVM (Fig. 1) and PVM lesions are observed in aged individuals^46^. Corpus callosum (CC) was used as a negative control, because we did not observe SUVR alterations in this white matter region (Fig. 1). Protein expression of HDACs (paralogs 1, 2, 3, and 6) relative to glyceraldehyde-3-phosphate dehydrogenase (GAPDH) were assessed by western blotting. Expression of HDAC1 and HDAC2, paralogs previously implicated in oligodendrocyte differentiation and myelination^24,47–49^, were significantly higher in older donors compared to younger donors in the PVM, whereas HDAC3 and HDAC6 were not different between groups (two-tailed unpaired *t*-test, false discovery rate (FDR) corrected for multiple comparisons; HDAC1: *P* = 0.022 and *P*_*FDR*_ = 0.031, HDAC2: *P* = 0.018 and *P*_*FDR*_ = 0.29, HDAC3: *P* = 0.62 and *P*_*FDR*_ = 0.81, HDAC6: *P* and *P*_*FDR*_ > 0.99, Fig. 3a,b, Supplementary Fig. 10). In the CC, none of the tested HDAC paralogs were significantly different between groups (two-tailed unpaired *t*-test, FDR corrected for multiple comparisons; HDAC1: *P* = 0.30 and *P*_*FDR*_ = 0.27, HDAC2: *P* = 0.15 and *P*_*FDR*_ = 0.28, HDAC3: *P* = 0.41 and *P*_*FDR*_ = 0.35, HDAC6: *P* = 0.73 and *P*_*FDR*_ = 0.70, Fig. 3c, Supplementary Fig. 11). Our post mortem HDAC expression data suggest that the observed increase in [^11^C]Martinostat uptake in the PVM is due to an increase in HDAC1 and HDAC2.

### SUVR shows sex-specific regional distribution

**Fig. 4:**
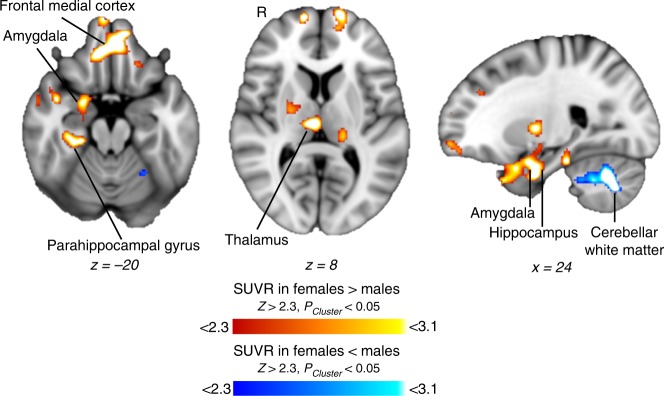
[^11^C]Martinostat uptake shows sex-specific differences. Voxel-wise comparison of SUVR between females (*n* = 20) and males (*n* = 21), controlled for age, total grey matter volume/intracranial volume, white matter volume/intracranial volume and cerebrospinal fluid volume/intracranial volume. Statistical maps were overlaid onto the MNI 1 mm template in radiological orientation (two-tailed unpaired *t*-test; *Z* > 2.3*, P*_*Cluster*_ < 0.05). Red–yellow represents regions significantly increased in females compared to males and blue-light blue represents regions significantly decreased in females compared to males

HDAC expression was compared between female (*n* = 20) and male (*n* = 21) subjects (Table 1, Supplementary Fig. 12a). Female and male subjects did not differ in age. Voxel-wise analysis of [^11^C]Martinostat SUVR was performed between groups controlling for age and brain volumes (*Z* score > 2.3, *P*_*cluster*_ < 0.05, Fig. 4, Supplementary Note 1, Supplementary Fig. 12b). SUVR was higher in females compared to males in the frontal medial cortex, amygdala, hippocampus, parahippocampal gyrus, and thalamus (Fig. 4). SUVR was lower in females compared to males in cerebellar white matter (Fig. 4). These results were recapitulated using a secondary normalization method (Supplementary Note 3, Supplementary Fig. 13).

**Table 1:** Subject demographic information

Characteristic	Females	Males
Number of subjects	20	21
Age range (years)	18–74	20–79
Age mean ± SD (years)	40.8 ± 18.1	40.7 ± 16.9
Body mass index mean ± SD	25.0 ± 4.0	24.5 ± 4.2
Injected dose mean ± SD (mCi)	5.1 ± 0.43	4.8 ± 0.65
Molar activity mean ± SD (mCi/nmol)	1.9 ± 0.72	1.9 ± 0.73

### SUVR may correlate with emotion regulation skills

To investigate the potential impact of changes in [^11^C]Martinostat uptake on human behavior, exploratory correlation analysis was performed between SUVR and cognition in a subset of subjects (*n* = 23, mean age ± standard deviation = 50 ± 17 years, 23–79-years-old, 12 females, 11 males) who underwent the Mayer–Salovey–Caruso Emotional Intelligence Test (MSCEIT)^50^. The MSCEIT is a socio-cognitive test that measures emotion regulation skills and social judgement. In a previous study, we determined that [^11^C]Martinostat SUVR in the dorsolateral prefrontal cortex was associated with the MSCEIT score, across subjects with schizophrenia and healthy controls^38^. Therefore, the MSCEIT score was chosen as the exploratory cognitive metric in this study. Preliminary voxel-wise analysis correlating SUVR with MSCEIT scores was performed while controlling for age, sex and brain volumes (*Z* score > 2.3, *P*_*cluster*_ < 0.05, Supplementary Fig. 14a). We observed a significant decrease in HDAC expression with MSCEIT scores in the cerebral white matter, specifically the inferior fronto-occipital fasciculus (IFOF) and the inferior longitudinal fasciculus (ILF) tracts, and the hippocampus (Supplementary Fig. 14a). Both the IFOF and ILF have previously been linked to multiple aspects of social cognition, including face perception and theory of mind^51,52^. In the hippocampus, HDACs negatively regulate the transcription of synaptic plasticity genes^19–21^, memory formation^14,21^ and mood-related social behaviors^53,54^ in rodents. Therefore, the hippocampus was selected for preliminary a priori ROI analysis and GTM partial volume correction was applied. In the human hippocampus, SUVR negatively correlated with MSCEIT scores (Spearman’s *r* = −0.43, *P* = 0.043, Supplementary Fig. 14b).

## Discussion

We discovered that HDACs, an epigenetic enzyme family with critical importance to neural processes, show age-related and sex-specific in vivo expression differences in the human brain. Previous studies relied on ex vivo measurements in preclinical rodent models, which have a shorter lifespan and more rapid sexual development compared to humans. Our work has the unique advantage of non-invasive assessment of HDACs throughout the living human brain.

Aging is the strongest risk factor for neurodegenerative diseases, such as Alzheimer’s disease and Parkinson’s disease^2,55^, and it contributes to pathophysiology in ways that are unresolved. We reveal through in vivo imaging, and validate through post mortem biochemistry, that HDAC1 and HDAC2 increase by as much as 30% in periventricular white matter across the adult lifespan. Interestingly, HDAC expression negatively correlates with age-associated alterations in white matter microstructure (gFA). Preliminary evidence also suggests that HDAC expression may negatively correlate with emotion regulation skills in the IFOF and ILF tracts, white matter areas linked to face processing, empathy, emotion recognition, and theory of mind^51,52^. We propose that these relationships could represent a mechanistic link between aging and perhaps cognitive performance, with neuroepigenetic regulation of genes that control white matter microstructural organization. More specifically, myelination may be implicated as HDAC1 and HDAC2 play key roles in remyelination in both the central and peripheral nervous systems^24,26,47,48,56^. Recent epigenomic analyses in post mortem brain tissues have identified distinct changes in histone acetylation and downstream gene transcription in neurodegenerative disease^13,57,58^, with a redistribution of epigenetic signatures observed between healthy aging and disease states^13,57^. Taken together with the increased HDAC2 expression observed in post mortem brain tissue from Alzheimer’s disease patients^20^, it is possible that HDAC1/2 accumulation in white matter may link aging to neurodegenerative disease through transcriptional dysregulation. Having determined the localization and magnitude of the HDAC1/2 increase, we are now poised to identify the specific cell-types involved and determine the downstream consequences of increased HDACs in the aging brain.

We observed a trend for a positive correlation between age and in vivo HDAC expression in the human hippocampus, whereas previous rodent studies reported significant age-related changes of HDAC expression^14,19^. It is possible that age-related changes are focal in subfields of the hippocampus such as CA1^15,18,42,59^, and are not detectable with our current methodology. We did observe novel sex-specific differences in HDAC expression throughout the limbic system, with higher HDAC expression in the subregions of the hippocampus, amygdala, and parahippocampus of females compared to males. Furthermore, SUVR in the hippocampus may negatively correlate with emotion regulation skills. Sexually dimorphic expression of HDAC could be relevant to the higher prevalence of neuropsychiatric disorders and neurodegenerative diseases such as major depression, generalized anxiety disorder, and Alzheimer’s disease, in females. Structural, functional, and neurochemical sexual dimorphisms including differences in dopamine, GABA, and serotonin neurotransmitter levels have been reported^60^. For example, in vivo expression of the serotonin receptor (5-HTR_1A_) is increased in the hippocampus and amygdala of females compared to males in humans^61,62^. Transcription of *5-HTR*_*1A*_ mRNA is regulated in part by HDAC1/2^63^, which suggests that altered HDAC levels could affect neurotransmitter systems. Additionally, serotonin-dependent activation of the serotonin receptor (5-HTR_2A_) inhibits *HDAC2* promoter activity in human cells^64^, while 5-HTR_2A_ inhibition increases *Hdac2* transcription and negatively affects synaptic remodeling and cognitive behavior through NF-κB signaling in rodents^65^, which may have relevance to depression and other neuropsychiatric disorders. Indeed, HDAC inhibitors elicit anti-depressive effects and alter social behavior in rodent models of neuropsychiatric disorders^54,64–68^. Moreover, in the hippocampus, HDACs also regulate the transcription of genes critical for synaptic plasticity^19–21^ and HDAC inhibition rescues memory deficits in rodent models of neurodegeneration^18,69,70^.

Due to the pleiotropic effects of HDACs on gene transcription, small changes in HDAC expression could have a large impact on neural circuits. For example, genetic knockdown of *Hdac2* expression by 25–30% increased transcription of memory-related genes and synaptic protein density, rescued long-term potentiation, and improved associative and spatial memory in an Alzheimer’s disease rodent model^20^. Furthermore, selective inhibition of HDAC1/2 modulated the transcription of over 1600 genes in the rodent brain^66^. These data support the hypothesis that the age-related and sex-specific in vivo HDAC expression patterns we observe in humans may influence neural function. The HDAC signal detected in each brain region, and each context (i.e. age or sex differences) may potentially correspond to a different subset of HDACs engaged by [^11^C]Martinostat. Previous work has demonstrated that [^11^C]Martinostat binds HDAC1, HDAC2, HDAC3, and to a lesser extent HDAC6^23,35^, thus we currently detect HDAC expression in aggregate rather than expression of individual paralogs. Therefore, as we translate paralog-selective HDAC imaging tools^71^ for human use, we will be better equipped to further interpret signal changes and relate each paralog to its respective phenotypes.

Our results suggest that in humans, age and sex are strongly associated with alterations in HDAC expression that may influence human behavior. As these biological variables contribute to disease risk, pharmacological agents such as HDAC inhibitors may represent a potential treatment to modify or intervene in pathophysiology.

## Methods

### Study design

Our main research objective was to compare [^11^C]Martinostat brain uptake across age and between sex in healthy adult subjects, using MR-PET. SUV normalized to whole brain mean (SUVR) from 60–90 min post radiotracer injection was the primary endpoint assessed. The study was approved by the Partners HealthCare Institutional Review Board (IRB) and the Massachusetts General Hospital (MGH) Radioactive Drug Research Committee. All subjects provided written informed consent according to the Declaration of Helsinki. Imaging procedures were performed at the Athinoula A. Martinos Center for Biomedical Imaging. See Table 1 for demographic information. Subjects underwent a physical examination by a licensed physician or nurse practitioner. Medical and medication history were recorded. Illicit and psychotropic drug use was assessed by a urine drug screen. A serum pregnancy test was performed for female subjects to ensure no pregnancy at the time of the scan. Forty-two subjects were scanned, but one subject was excluded from primary analyses for a dental work-related artifact. Diffusion images were available to assess white matter microstructure in forty subjects. MSCEIT^50^ scores were available to assess cognitive performance in twenty-three subjects; scores were age- and sex-corrected. We furthered our findings through biochemical analysis of post mortem human brain tissue from eighteen donors, to measure protein expression of HDAC paralogs. Imaging and biochemical studies were not blinded, and no outliers were excluded.

### Subject inclusion/exclusion criteria

Subjects were healthy as determined by a licensed physician/nurse practitioner. Subjects had no history of psychiatric or major physical illness (including but not limited to depression, schizophrenia, mild cognitive impairment, diabetes mellitus, or head trauma). Subjects had no present substance abuse. Subjects who were taking psychotropic medications, or using illicit drugs or marijuana, were excluded. Subjects were excluded if they met any MR-PET safety contraindications, including pregnancy or breastfeeding.

### Radiosynthesis of [^11^C]Martinostat

See Supplementary Methods.

### MR-PET data acquisition

See Supplementary Methods.

### MR data analysis

See Supplementary Methods.

### Primary PET image analysis

Primary analysis was performed in 41 subjects (18–79-years-old, 20 females, 21 males). Motion correction was applied with the MCFLIRT tool^72^ in FSL version 5.0.7^73^. Motion-corrected SUV frames from 60–90 min were averaged to create a mean SUV image for each subject. SUV images were registered to the subject’s MEMPRAGE using mri_coreg from FreeSurfer 6.0, skull-stripped, and resampled to 2-mm isotropic voxel size. The subject’s MEMPRAGE was registered to MNI space using linear FLIRT^72^ and nonlinear FNIRT^74^ algorithms in FSL. The registration matrix was then applied to move SUV images into MNI space. SUV images in native space and MNI space were (1) intensity normalized to whole brain mean as SUVR^38–40^ and (2) intensity normalized to mean pons SUV as SUVR_Pons_, using individual native space pons masks generated by FreeSurfer’s automated brainstem segmentation^75^. SUVR and SUVR_Pons_ images were spatially smoothed 8 mm full width at half maximum (FWHM). Whole brain voxel-wise analysis was performed with smoothed SUVR and SUVR_Pons_ images in MNI space using FSL’s randomise^76^ and FEAT^77^. Region of interest (ROI) analysis was performed with unsmoothed SUVR images in native space using masks for the hippocampus and cerebral white matter generated by FreeSurfer’s automated parcellation and segmentation^78^. ROI analysis was also performed with unsmoothed SUVR images in native space using eroded cerebral white matter masks.

### Partial volume correction

Partial volume correction was applied using RBV analysis and ROI based GTM, both available in PETSurfer^79^.

### Quantitative analysis using kinetic modeling

An arterial line (A-line) was placed in a subset of subjects (*n* = 7, 22–53-years-old, two females, five males) in order to perform full quantitative analysis to determine distribution volume (*V*_T_). The A-line was placed in the radial artery by a licensed anesthesiologist for arterial blood sampling to determine plasma radioactivity and radioactive metabolites. Blood samples were drawn by an experienced nurse practitioner. Arterial plasma and metabolite analyses were performed. PET images were reconstructed with the following scheme (10 s × 8, 20 s × 3, 30 s × 2, 60 s × 1, 120 s × 1, 180 s × 1, 300 s × 8, and 600 s × 4) and reconstructed in units of both bqml and SUV. Kinetic modeling was executed with PMOD version 3.4 (PMOD Technologies Ltd., Zurich, Switzerland). A 2TCM was applied to the regional time-activity curves (TACs) extracted from volumes of interest (VOIs), and the metabolite-corrected arterial plasma was used as the input function to derive *V*_T_. VOIs were defined according to the Automated Anatomical Labeling (AAL) human brain atlas distributed with PMOD^80^. As done previously, we included fourteen VOIs with bilateral VOIs merged together^23^. The VOIs included composite VOIs for the frontal, parietal, temporal and occipital lobes, as well as VOIs for the insula, cingulate, caudate, putamen, pallidum, thalamus, hippocampus, amygdala, cerebellum, and white matter. SUV from 60 to 90 min post radiotracer injection was normalized to whole brain mean (SUVR). SUV from 60 to 90 min post radiotracer injection was also normalized to mean pons SUV (SUVR_Pons_). *V*_T_, SUVR, and SUVR_Pons_ images were spatially smoothed with an 8 mm FWHM gaussian kernel. Pearson correlation was then conducted between SUVR (or SUVR_Pons_) and *V*_T_ values for the fourteen VOIs, for each subject.

### Post mortem tissue analysis

Human brain tissue was obtained from the National Institutes of Health (NIH) NeuroBioBank, specifically the Harvard Brain Tissue Resource Center and the University of Maryland Brain and Tissue Bank. Informed consent was obtained from next of kin for all donors. Donor brains had a neuropathology diagnosis of normal. Periventricular white matter or corpus callosum was homogenized in lysis buffer containing PBS, 0.15% NP-40, and a protease inhibitor cocktail (Roche #11836170001) at 50 mg/ml. Homogenate was sonicated at 50% power (Fisher Scientific Model #CL-18) on ice for thirty pulses of 1 s duration followed by 2 s of rest. Tissue lysate was plunged through a 23-gauge syringe 10 times, incubated with rotation at 4 °C for 20 min, and centrifuged 18,000×*g* at 4 °C for 20 min. Supernatant was collected and total protein concentration was measured using a bicinchoninic acid (BCA) protein assay (Pierce #23227). Lysate (12 µg per sample) was separated with Criterion Stain-Free 4–20% gels (Biorad #567-8095) and transferred to PVDF membrane (Biorad #162-0264). Membranes were blocked in 5% milk (Biorad #170-6404) prepared in tris buffered saline with 0.1% Tween 20 (TBST) overnight at 4 °C. Remaining procedures were performed at room temperature. Membranes were washed in TBST and incubated with primary antibodies in TBST containing 1% milk for 90 min (HDAC1: Thermo Fisher #PA1-860 at 1:6000, HDAC2: Abcam #ab124974 at 1:5000, HDAC3: Abcam #ab32369 at 1:2500, HDAC6: Santa Cruz #sc11420 at 1:6000, GAPDH: Abcam ab8245 at 1:2000000). Membranes were washed in TBST and incubated with secondary antibodies in TBST containing 1% milk for 60 min (anti-rabbit-HRP: Cell signaling #7074S at 1:5000, anti-mouse HRP: Cell Signaling #7076S at 1:5000). Membranes were washed in TBST, developed with ECL reagent (GE Healthcare #RPN2232), and visualized with a Chemidoc XRS imager (Biorad #170-8265). For analysis, western blot images were converted from Image lab 5.2.1 files (.scn) to 600 dpi and saved as Tif files. The images were analyzed in Image J after conversion to 8-bit, subtraction of background with a 50-pixel rolling ball radius, and inversion. Mean band intensity was quantified using the measurement tool. See Supplementary Figs10. and 11 for uncropped western blot images.

### Statistical analysis

FSL was used for voxel-wise analyses using FEAT or randomise. For all other analyses, GraphPad Prism version 8.01 was used.

For voxel-wise comparisons (Figs. 1 and 2, Supplementary Figs. 4a, 7a, b, and 13b), FSL’s randomise was used with threshold-free cluster enhancement (TFCE) and 10,000 permutations, with family wise error rate (FWE) to correct for multiple comparisons (*P*_*FWE*_ < 0.05). For voxel-wise comparisons (Fig. 4, Supplementary Figs. 13a and 14a), FSL’s FEAT with ordinary least squares (OLS) mixed-effects modeling was used with *Z* score > 2.3 and *P*_*cluster*_ < 0.05. Voxel-wise analyses were conducted with the following regressors: (1) age vs. SUVR: sex, total grey matter volume/intracranial volume (ICV), white matter volume/ICV and cerebrospinal fluid volume/ICV; (2) age vs. SUVR (within WM using region-based voxel-wise partial volume correction), age vs. SUVR_Pons_, as well as age vs. gFA: sex; (3) sex difference in SUVR: age, total grey matter volume/ICV, white matter volume/ICV and cerebrospinal fluid volume/ICV; (4) sex difference in SUVR_Pons_: age; (5) MSCEIT T-scores vs. SUVR: age, sex, total grey matter volume/ICV, white matter volume/ICV and cerebrospinal fluid volume/ICV.

For ROI analyses, (1) Pearson correlations to relate (*V*_T_ to SUVR or SUVR_Pons_; (2) Spearman rank-order correlations to relate age with SUVR or SUVR_Pons_, age with brain volumes, SUVR with BMI, SUV in the whole brain with SUV in the pons, gFA with SUVR, and MSCEIT T-scores with SUVR; (3) two-tailed unpaired Mann–Whitney *U*-test to assess differences in age or brain volumes between females and males.

For biochemical analyses, two-tailed unpaired *t*-tests comparing individual HDAC paralog expression between older and younger donor tissues with FDR correction for multiple comparisons.

### Reporting summary

Further information on research design is available in the Nature Research Reporting Summary linked to this article.

## Supplementary information

Supplementary Information

Reporting Summary

## Data Availability

The data that support these findings are available from the corresponding author, J.M.H., upon reasonable request. Human subject data will be deidentified to protect confidentiality.

## Code availability

Custom codes for MR and PET data processing are available from the corresponding author, J.M.H., upon reasonable request.
