# Supplementary material for: Neuroepigenetic signatures of age and sex in the living human brain
Source: Nat Commun. 2019 Jul 3;10:2945. doi: 10.1038/s41467-019-11031-0 (PMC6610136; doi:10.1038/s41467-019-11031-0)
Supplement: Supplementary file 1 — Supplementary Information [file 41467_2019_11031_MOESM1_ESM.pdf]

Supplementary Information

Neuroepigenetic signatures of age and sex in the living human brain

Gilbert and Zürcher *et al.*

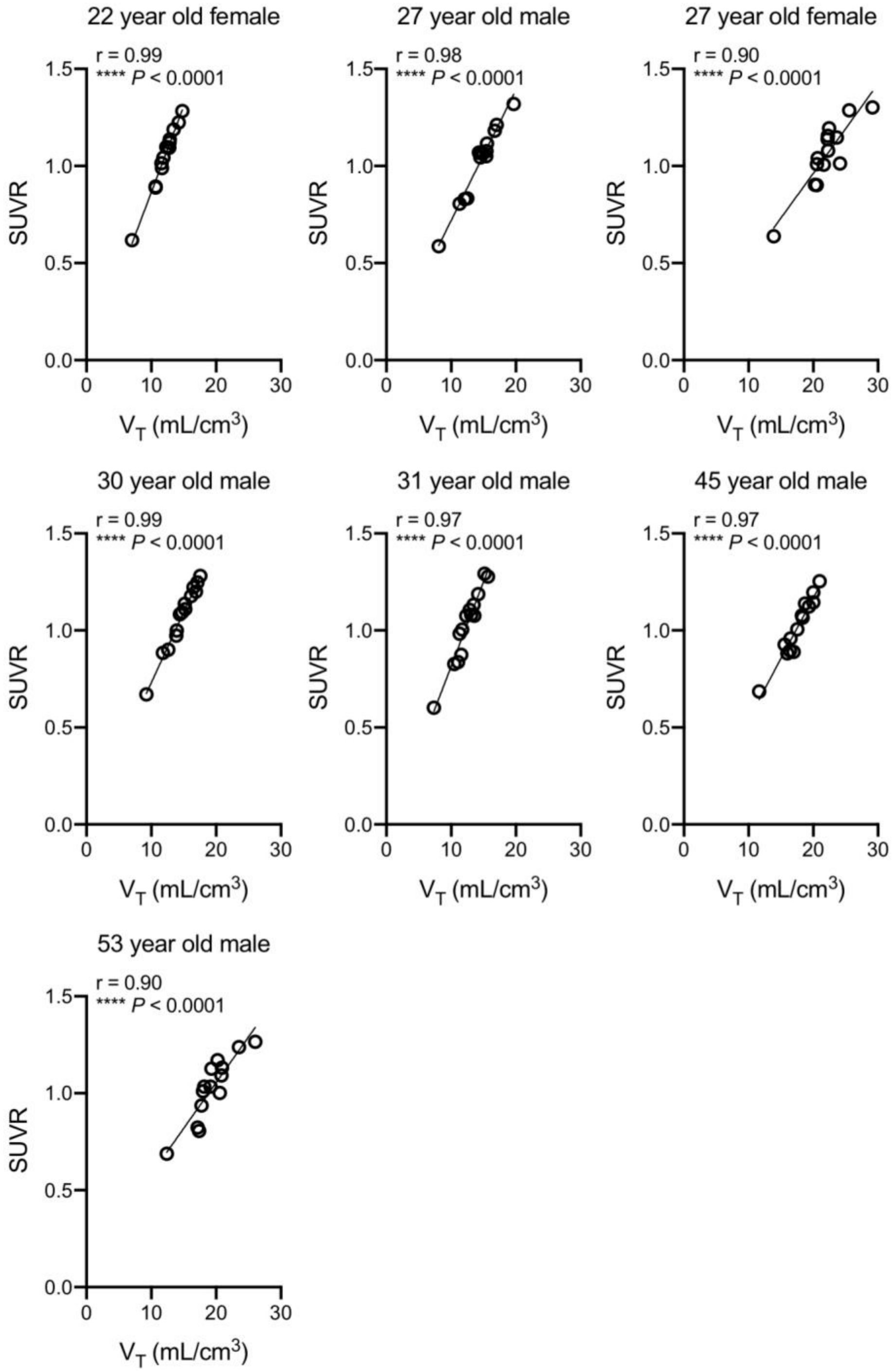

Supplementary Figure 1. [^{11}C]Martinostat SUVR is an appropriate surrogate measure for V_T. Distribution volume (V_T) time activity curves (TACs) were derived from a two-tissue compartmental model (2TCM) using metabolite-corrected arterial plasma as an input function. Pearson correlation analysis was performed between regional V_T values and SUVR values for each subject (*n*=7). Each circle represents a separate brain region (*n*=14 brain regions).

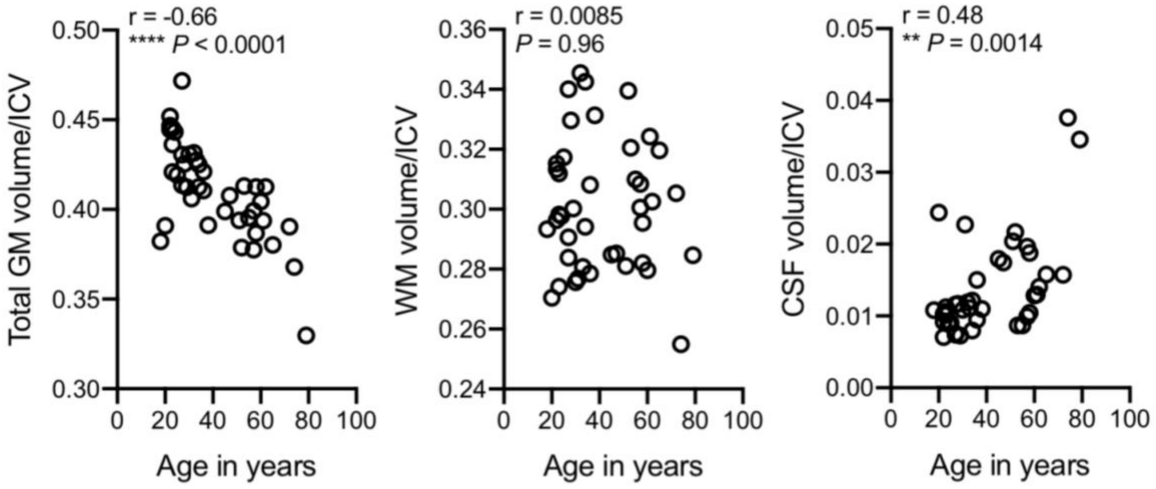

Supplementary Figure 2. Volumetric changes with age. Spearman correlation analysis between age and total gray matter volume/intracranial volume, white matter volume/intracranial volume and cerebrospinal fluid volume/intracranial volume (*n*=41).

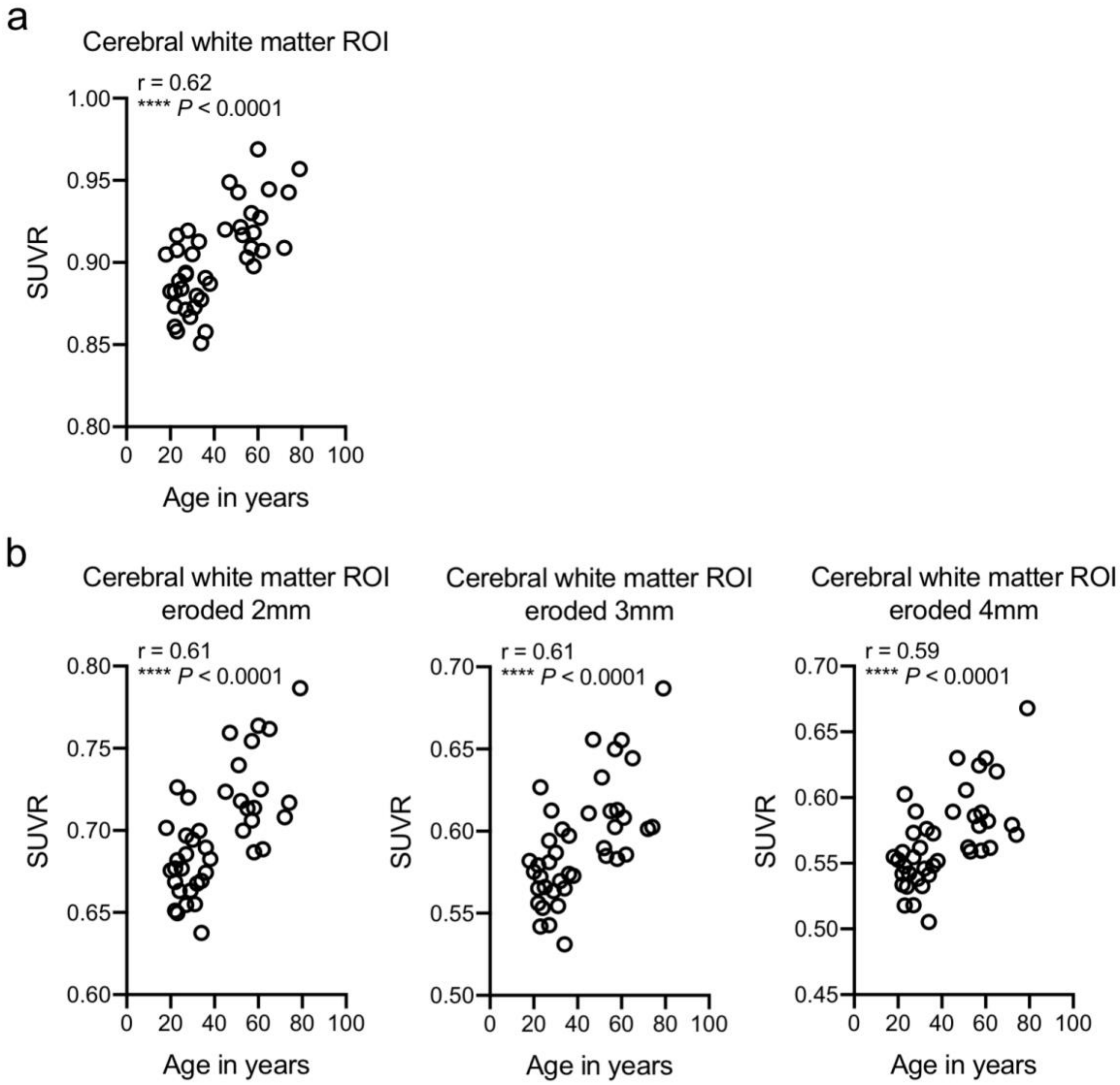

Supplementary Figure 3. [^{11}C]Martinostat uptake increases with age in the white matter in the native space ROI and eroded masks. **a** Spearman correlation analysis between age and SUVR extracted from cerebral white matter based on automated FreeSurfer segmentation in native space (*n*=41). **b** Spearman correlation analysis between age and SUVR extracted from cerebral white matter based on automated FreeSurfer segmentation in native space using masks eroded with a 2mm sphere, 3mm cube or 4mm sphere 3D kernel (*n*=41).

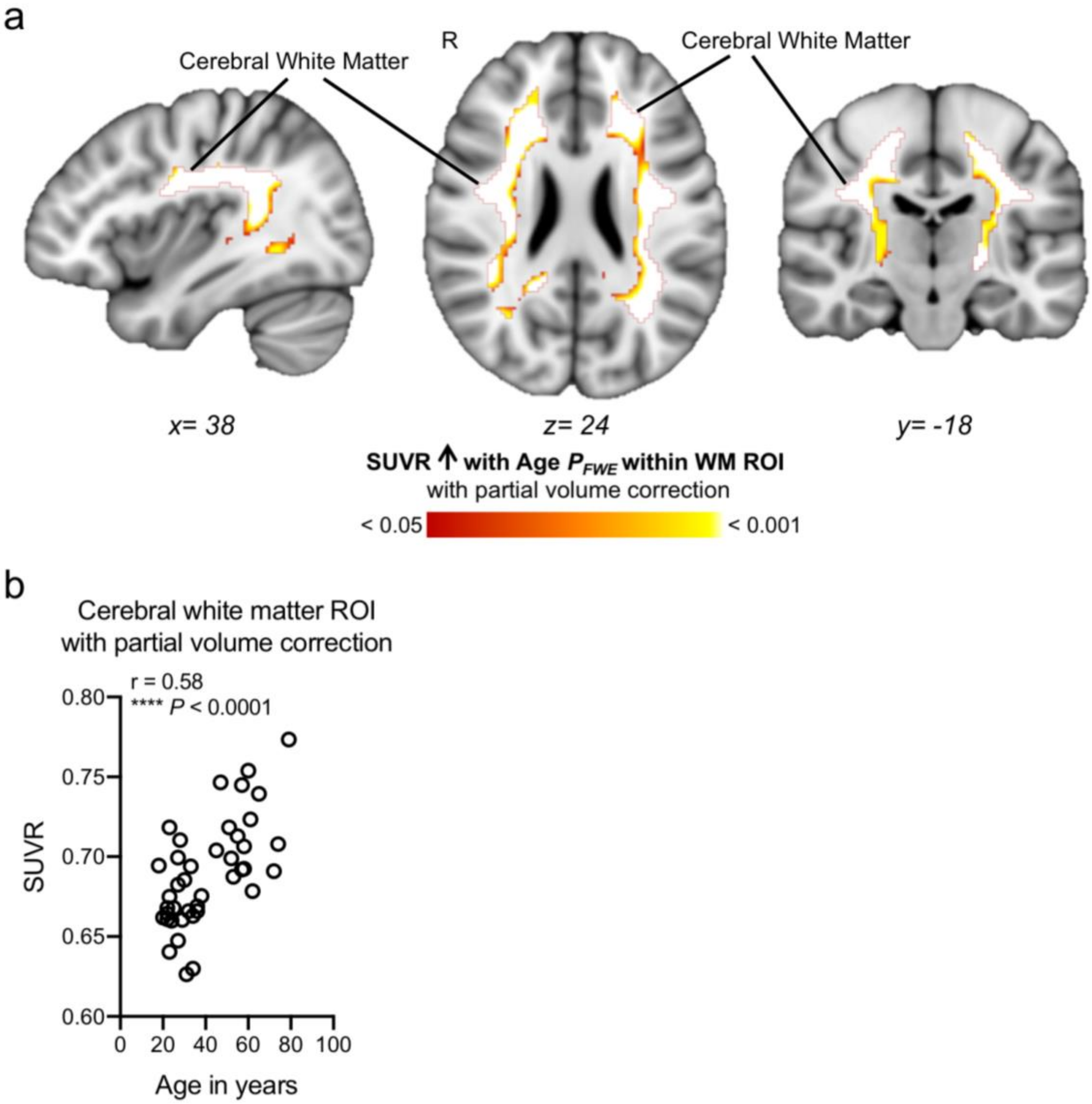

Supplementary Figure 4. [11C]Martinostat uptake increases with age in the white matter after partial volume correction, supporting the non-partial volume corrected findings. **a** Voxel-wise correlations of SUVR with age, controlled for sex (*n*=41) were restricted to white matter and region-based voxel-wise correction was applied to correct for partial volume effects. Statistical maps were overlaid onto the MNI 1mm template in radiological orientation (family wise error rate (FWE) corrected for multiple comparisons, $P_{FWE} < 0.05$; non-parametric permutation testing *n*=10,000 permutations). Red-yellow represents regions significantly increased with age. **b** SUVR was extracted from cerebral white matter based on automated FreeSurfer segmentation in native space, using the geometric transfer matrix method to correct for partial volume effects. Spearman correlation analysis between age and SUVR (*n*=41).

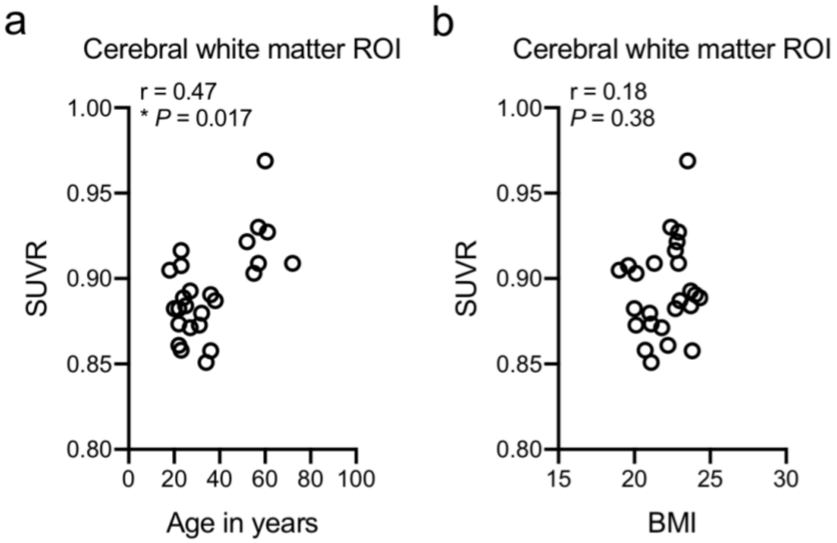

Supplementary Figure 5. [^{11}C]Martinostat uptake increases with age are not driven by body mass index (BMI). **a** Spearman correlation analysis between age and SUVR extracted from cerebral white matter, based on automated FreeSurfer segmentation in native space, in subjects with a normal BMI of 18-25 (*n*=25). **b** Spearman correlation analysis between BMI and SUVR extracted from cerebral white matter, based on automated FreeSurfer segmentation in native space, in subjects with a normal BMI of 18-25 (*n*=25).

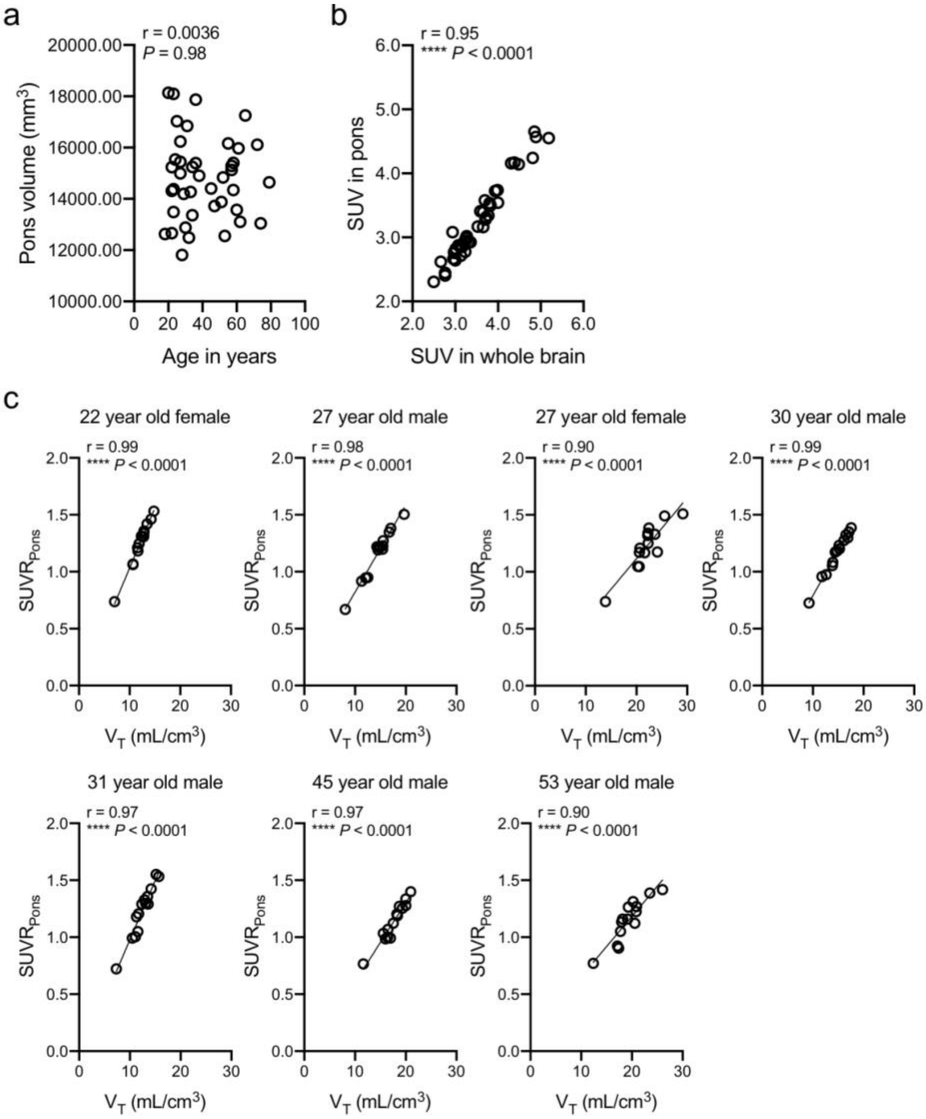

Supplementary Figure 6. Pons characteristics. **a** Spearman correlation analysis between age and pons volume, based on automated FreeSurfer segmentation in native space (*n*=41). **b** Spearman correlation analysis between SUV extracted from the whole brain and SUV extracted from the pons, based on automated FreeSurfer segmentation in native space (*n*=41). **c** Distribution volume (V_T) time activity curves (TACs) were derived from a two-tissue compartmental model (2TCM) using metabolite-corrected arterial plasma as an input function. Individual subject SUV maps were normalized to mean SUV from the pons as $SUVR_{Pons}$. Pearson correlation analysis was performed between regional V_T values and $SUVR_{Pons}$ values for each subject (*n*=7). Each circle represents a separate brain region (*n*=14 brain regions).

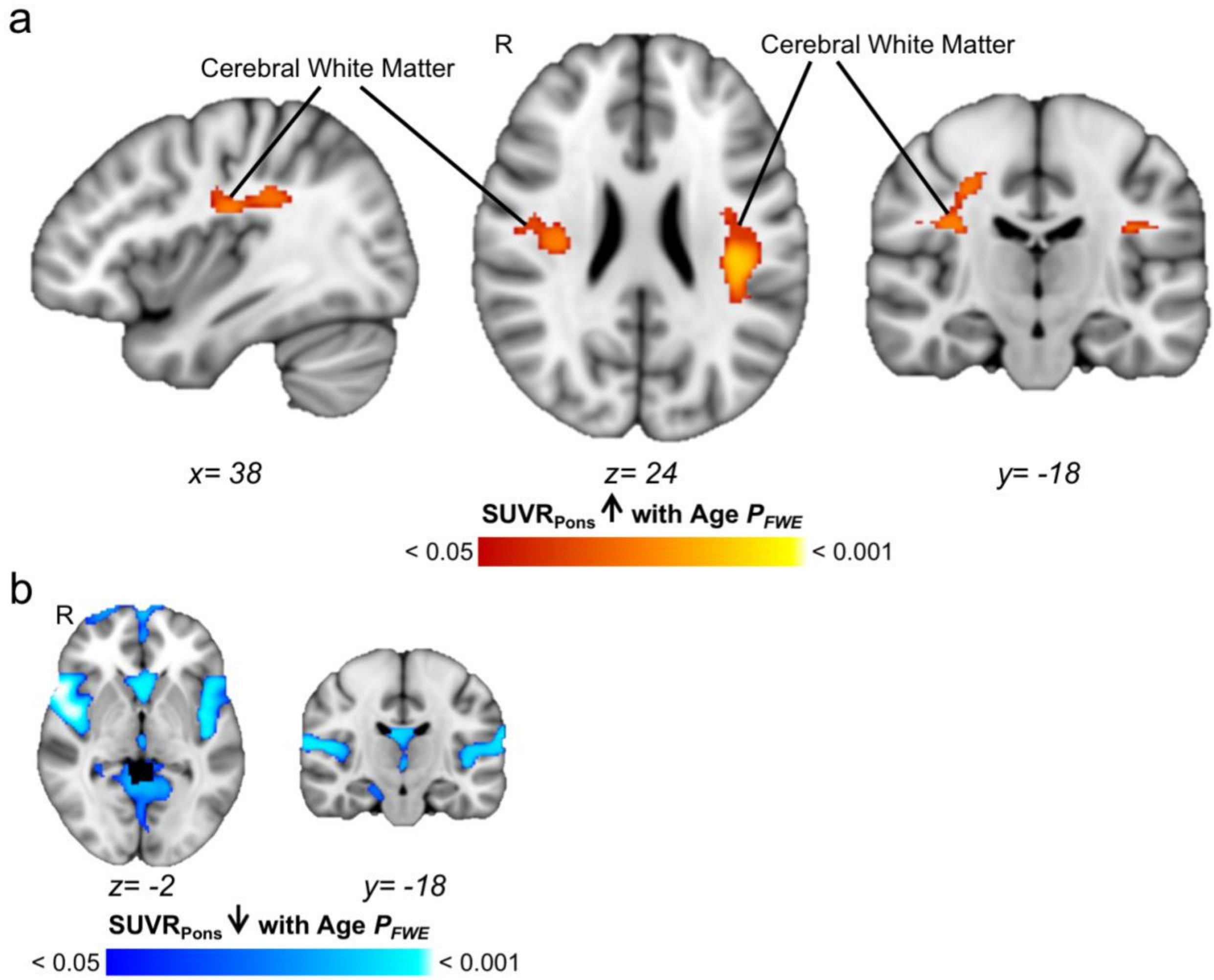

Supplementary Figure 7. [^{11}C]Martinostat uptake increases with age in the white matter using an alternative normalization method. Individual subject SUV maps were normalized to mean SUV from the pons as $SUVR_{Pons}$. Voxel-wise correlations of $SUVR_{Pons}$ with age, controlled for sex (n=41). Statistical maps were overlaid onto the MNI 1mm template in radiological orientation (family wise error rate (FWE) corrected for multiple comparisons, $P_{FWE} < 0.05$; non-parametric permutation testing n=10,000 permutations). **a** Red-yellow represents regions significantly increased with age and **b** blue-light blue represents regions significantly decreased with age.

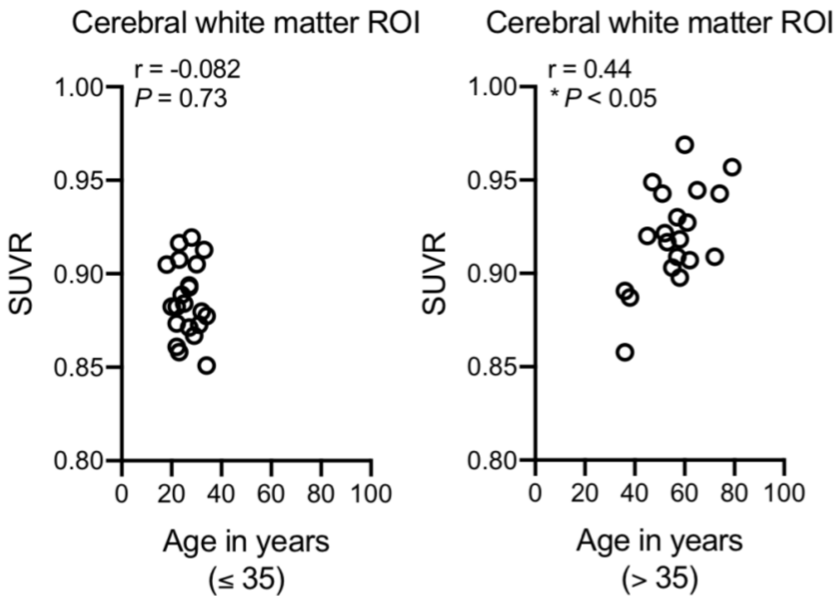

Supplementary Figure 8. [^{11}C]Martinostat uptake increases with age in the white matter in mid to late adulthood. Spearman correlation analysis between age and SUVR extracted from cerebral white matter based on automated FreeSurfer segmentation in native space was performed with subjects split at the median age of 35 (*Left*, *n*=20 subjects 18-35 years old; *Right*, *n*=21 subjects 36-79 years old).

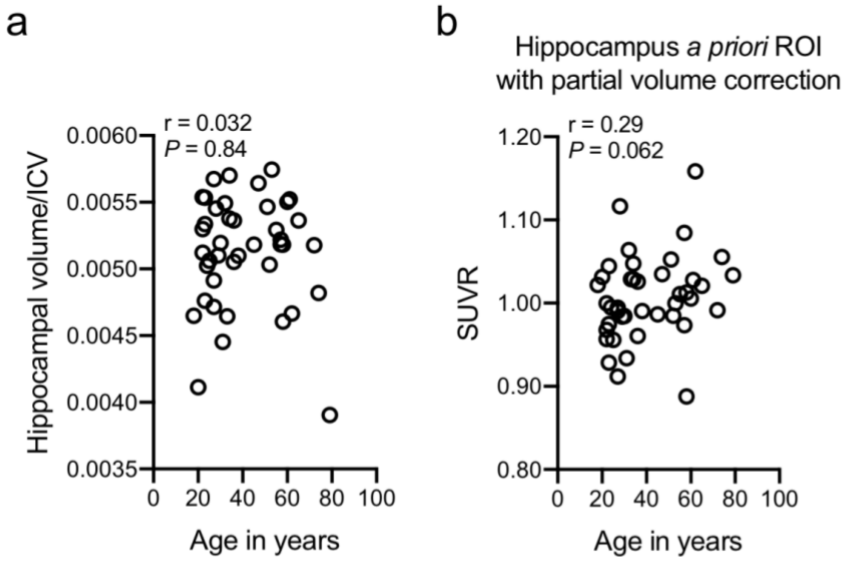

Supplementary Figure 9. [^{11}C]Martinostat uptake in the hippocampus does not correlate with age but indicates a trend. **a** Spearman correlation analysis between age and hippocampal volume/intracranial volume (*n*=41). **b** SUVR was extracted from the hippocampus *a priori* ROI, based on automated FreeSurfer segmentation in native space, using the geometric transfer matrix method to correct for partial volume effects. Spearman correlation analysis between age and SUVR (*n*=41).

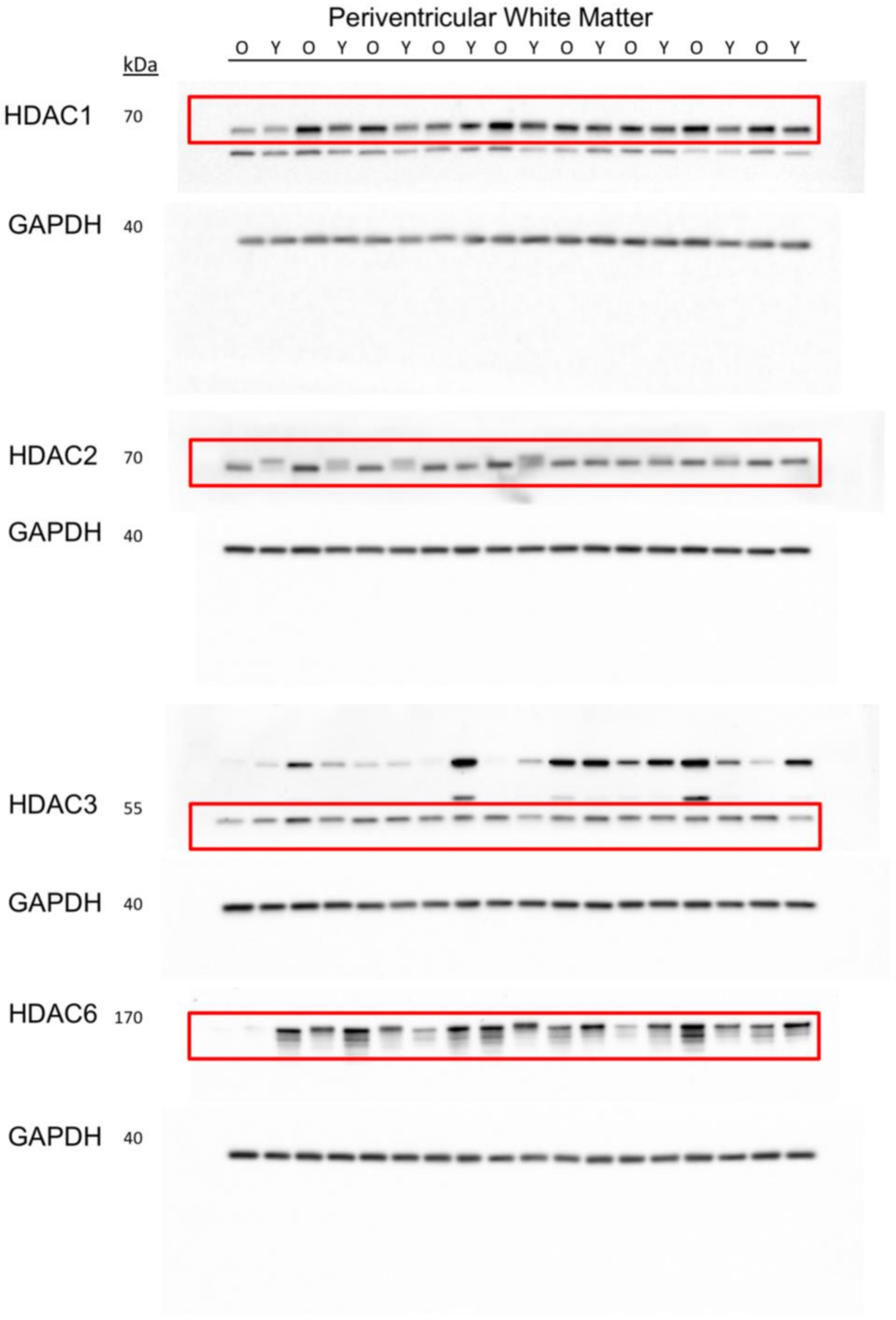

Supplementary Figure 10. HDAC1 and HDAC2 expression is increased in the periventricular white matter of elderly donors. Whole-cell lysates were prepared from post mortem periventricular white matter. Protein expression of HDAC paralogs (1, 2, 3, and 6) were compared between younger donors (*n*=9, 16-21 years old) and older donors (*n*=9, 76-100 years old) by western blotting. Uncropped western blot images accompany Fig. 3.

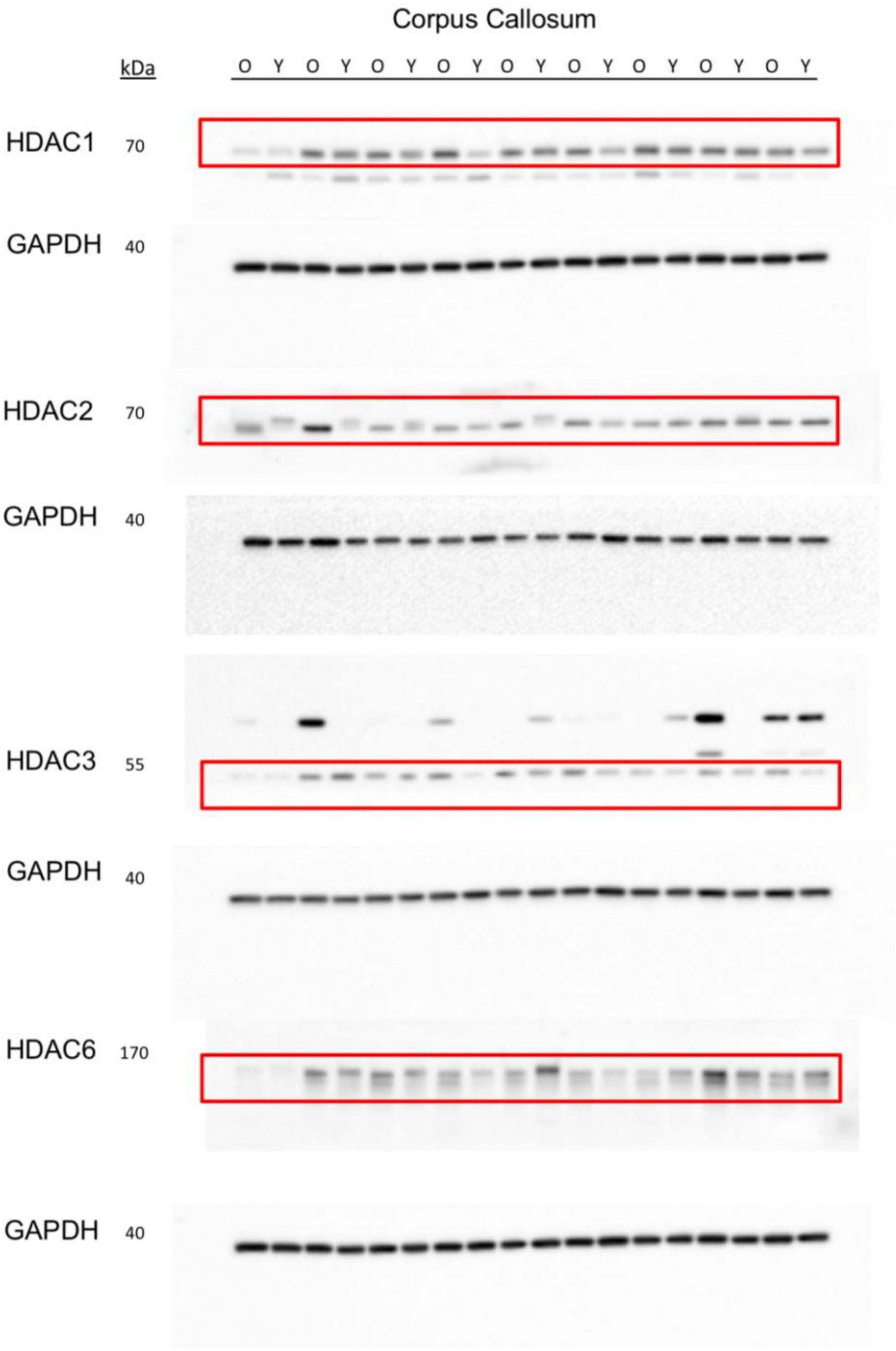

Supplementary Figure 11. HDAC1 and HDAC2 expression is not altered in the corpus callosum of elderly donors. Whole-cell lysates were prepared from post mortem corpus callosum. Protein expression of HDAC paralogs (1, 2, 3, and 6) were compared between younger donors (*n*=9, 16-21 years old) and older donors (*n*=9, 76-100 years old) by western blotting. Uncropped western blot images accompany Fig. 3.

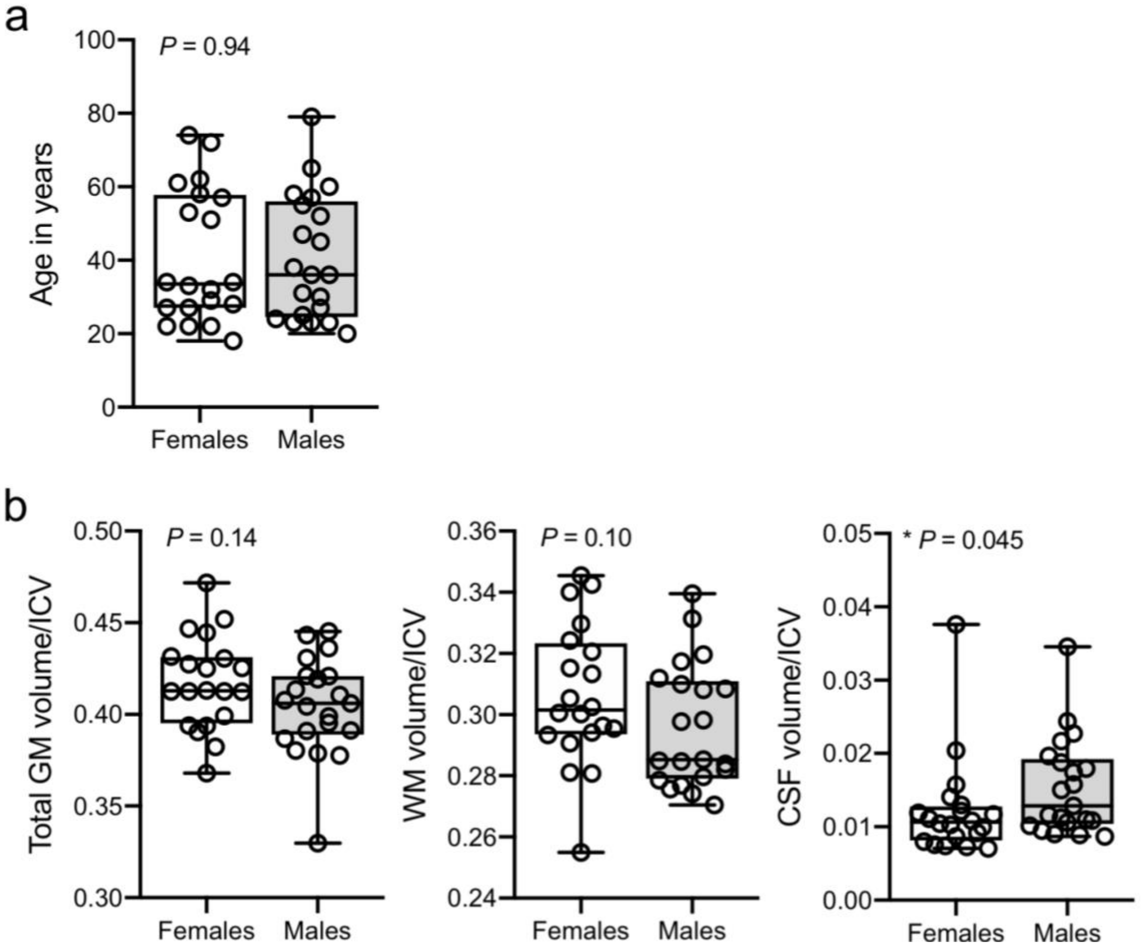

Supplementary Figure 12. Age and brain volumes between sexes. **a** Boxplots (median, first quartile, third quartile, range of min-max) depict the age of females (*n*=20) and males (*n*=21) (two-tailed unpaired Mann Whitney *U*-test). **b** Boxplots (median, first quartile, third quartile, range of min-max) depict the total gray matter volume/intracranial volume, white matter volume/intracranial volume or cerebrospinal fluid volume/intracranial volume from females (*n*=20) and males (*n*=21) (two-tailed unpaired Mann Whitney *U*-test).

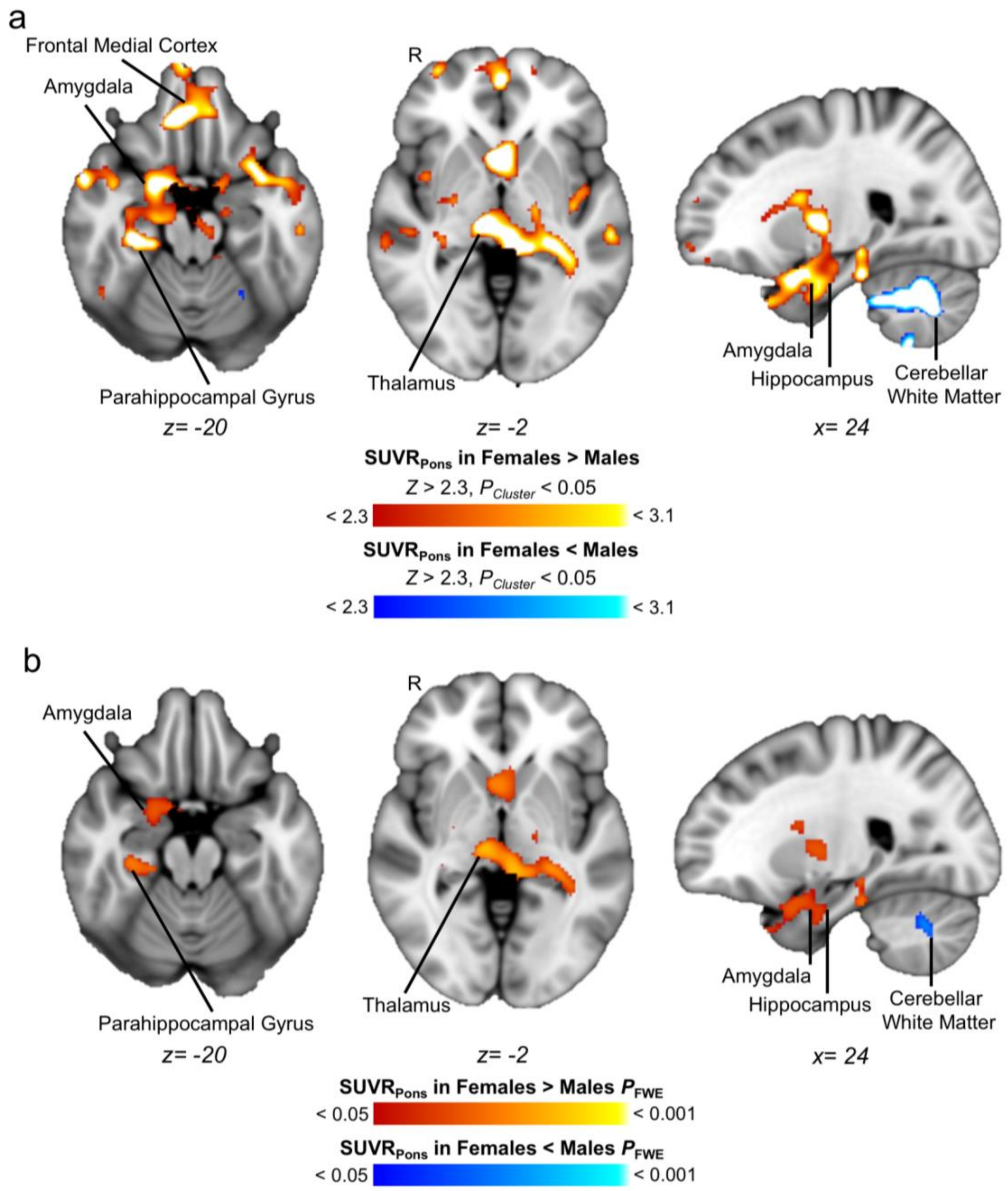

Supplementary Figure 13. [^{11}C]Martinostat uptake shows sex-specific differences using an alternative normalization method. Individual subject SUV maps were normalized to mean SUV from the pons as $SUVR_{Pons}$. Voxel-wise comparison of $SUVR_{Pons}$ between females (*n*=20) and males (*n*=21), controlled for age. **a** Statistical maps were overlaid onto the MNI 1mm template in radiological orientation (two-tailed unpaired *t*-test; $Z > 2.3$, $P_{Cluster} < 0.05$). Red-yellow represents regions significantly increased in females compared to males and blue-light blue represents regions significantly decreased in females compared to

males. **b** Statistical maps were overlaid onto the MNI 1mm template in radiological orientation (two-tailed unpaired *t*-test; family wise error rate (FWE) corrected for multiple comparisons, $P_{FWE} < 0.05$; non-parametric permutation testing n=10,000 permutations). Red-yellow represents regions significantly increased in females compared to males and blue-light blue represents regions significantly decreased in females compared to males.

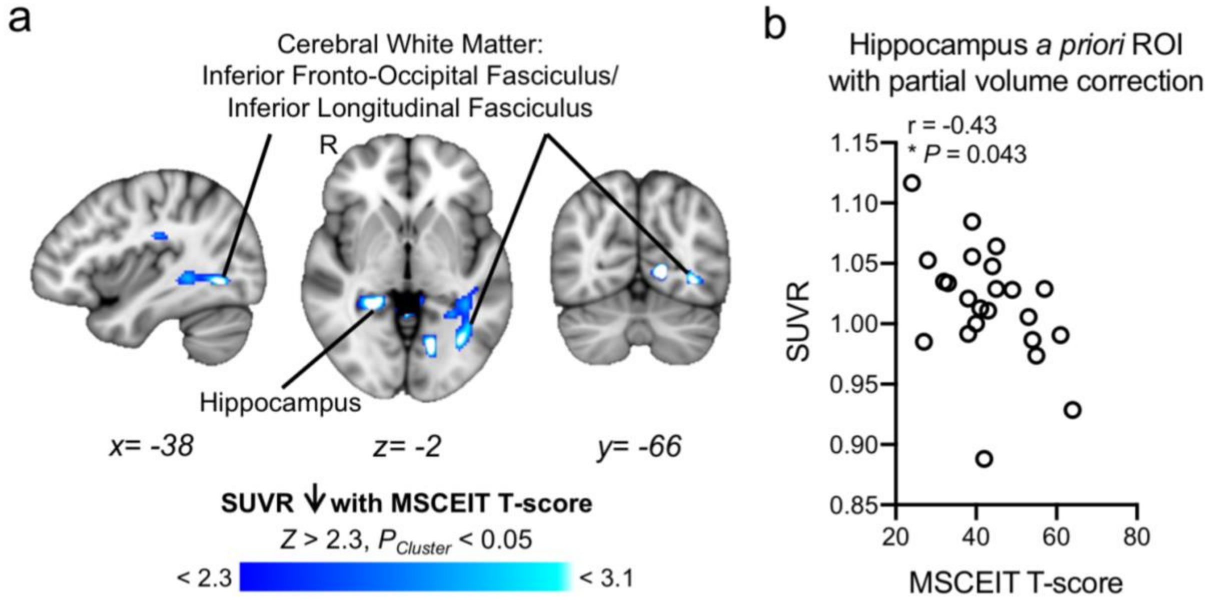

Supplementary Figure 14. [^{11}C]Martinostat uptake negatively correlates with emotion regulation skills/social judgment assessed by MSCEIT. **a** Voxel-wise correlations of SUVR with Mayer-Salovey-Caruso Emotional Intelligence Test (MSCEIT) T-scores, controlled for age, sex, total gray matter volume/intracranial volume, white matter volume/intracranial volume and cerebrospinal fluid volume/intracranial volume (n=23). Statistical maps were overlaid onto the MNI 1mm template in radiological orientation (Z> 2.3, $P_{Cluster}$< 0.05). Blue-light blue represents regions significantly decreased with MSCEIT T-scores. **b** SUVR was extracted from the hippocampus *a priori* ROI, based on automated FreeSurfer segmentation in native space, using the geometric transfer matrix method to correct for partial volume effects. Spearman correlation analysis between MSCEIT T-scores and SUVR (n=23).

Supplementary Note 1. Brain volumes. It is well-established that regional brain volumes are altered in normal aging[1]. As expected, total gray matter (GM) volume normalized to intracranial volume (ICV) decreased with age (Spearman's r= -0.66, P< 0.0001) and cerebrospinal fluid (CSF) volume normalized to ICV increased with age (Spearman's r= 0.48, P= 0.0014, **Supplementary Figure 2**) in subjects imaged in this study (n= 41). No statistically significant difference was observed in white matter (WM) volume normalized to ICV with age (Spearman's r= 0.0085, P= 0.96, **Supplementary Figure 2**). CSF volume normalized to ICV was higher in males than females (P= 0.045, **Supplementary Figure 12b**). As SUVR values could be affected by changes in brain volume, total GM normalized to ICV, WM normalized to ICV, and CSF normalized to ICV were added as regressors of non-interest to all SUVR whole brain voxel-wise analyses.

Supplementary Note 2. Body mass index. To support that the age-related [^{11}C]Martinostat uptake differences observed in our study were not driven by body mass index (BMI), we assessed [^{11}C]Martinostat uptake in subjects within a normal BMI range of 18-25 (n= 25). As expected, age was positively correlated with HDAC expression in cerebral white matter (Spearman's r= 0.47, P= 0.017, **Supplementary Figure 5a**), but BMI was not correlated with HDAC expression in this group of subjects (r= 0.18, P= 0.38, **Supplementary Figure 5b**).

Supplementary Note 3. Pons normalization. To further verify that [^{11}C]Martinostat uptake differences were not driven by age-related brain atrophy, the pons was chosen as a secondary normalization region. Pons volume did not change with age in subjects imaged in this study (Spearman's r= 0.0036, P= 0.98, **Supplementary Figure 6a**). SUV in the pons was strongly associated with SUV in the whole brain (Spearman's r= 0.95, P< 0.0001, **Supplementary Figure 6b**). We determined that standard uptake value (SUV) normalized to pons mean ($SUVR_{Pons}$) from 60-90 min post radiotracer injection is an appropriate surrogate measure for the distribution volume (V_T) in a subset of healthy young and old adults. Regional $SUVR_{Pons}$ were strongly correlated with V_T values derived from a two-tissue compartmental model

(2TCM), using metabolite-corrected arterial plasma as an input function (all Pearson's r≥ 0.90, all P< 0.0001, **Supplementary Figure 6c**). Voxel-wise analysis correlating $SUVR_{Pons}$ with age was performed while controlling for sex. $SUVR_{Pons}$ was increased with age throughout the cerebral white matter, recapitulating our primary finding (P_{FWE}< 0.05, **Supplementary Figure 7a**). In addition to regional increases, voxel-wise analysis also showed decreased $SUVR_{Pons}$ with age limited to the CSF, and small portions of opercular and insular cortices adjacent to the CSF, which were likely driven by age-associated partial volume effects. (P_{FWE}< 0.05, **Supplementary Figure 7b**).

Pons normalization was also used to verify that [^{11}C]Martinostat uptake differences were not driven by sex-related differences in brain volume. Voxel-wise analysis of [^{11}C]Martinostat $SUVR_{Pons}$ was performed between groups controlling for age (Z score >2.3, $P_{cluster}$< 0.05, **Supplementary Figure 13a**) and recapitulated our primary findings. $SUVR_{Pons}$ was higher in females compared to males in the frontal medial cortex, amygdala, hippocampus, parahippocampal gyrus, and thalamus (**Supplementary Figure 13a**). $SUVR_{Pons}$ was lower in females compared to males in cerebellar white matter (**Supplementary Figure 13a**). This finding was recapitulated for the majority of the brain regions using non-parametric analysis and a statistical threshold of P_{FWE}< 0.05 (**Supplementary Figure 13b**).

Supplementary Methods

Radiotracer synthesis according to cGMP guidelines, as detailed in a previous publication[2]. [^{11}C]CO_2 was obtained via the ^{14}N (p, α) ^{11}C reaction with 11 MeV protons (Siemens Eclipse cyclotron). [^{11}C]CO_2 was then trapped in a TRACERlab FX-MeI synthesizer (General Electric). [^{11}C]CH_4 was obtained by the reduction of [^{11}C]CO_2 in the presence of Ni/hydrogen at 350°C and recirculated through an oven containing I_2 to produce $^{11}CH_3I$. [^{11}C]Martinostat was synthesized using 2 mg of the desmethyl precursor in 300 μL of DMSO using a TRACERlab FX-M synthesizer. [^{11}C]MeI was introduced into the vessel, which was sealed and heated to 100°C for 5 min. The solution was then diluted with 1.2 mL of water and loaded onto a semi-preparative HPLC (Agilent Eclipse XDB-18 column, 5 μm, 9.4x250 mm^2. PN: 990967-202). Purification using this column was achieved with 40% 0.01M NaOH / 60% EtOH HPLC as

the mobile phase (2 ml/min, 254 nm). [^{11}C]Martinostat was collected from the HPLC into a sterile and pyrogen-free glass bulb that was pre-charged with sterile water (14.3 ml), saline (0.7 ml, 23.4% sodium chloride), and 2 N HCl (9 μL). Chemical and radiochemical purities were ≥ 95 %.

MR-PET data acquisition. [^{11}C]Martinostat (*n*=41 subjects, mean dose± standard deviation= 4.96±0.58 mCi) was injected as a manual bolus by a licensed nuclear medicine technologist through an intravenous catheter in the antecubital vein of the arm. MR and PET images were simultaneously acquired on a 3T Siemens TIM Trio containing a BrainPET insert[3]. An eight-channel MR head coil was used. A T1-weighted multi-echo MPRAGE sequence (MEMPRAGE) with epi navigator[4,5] [repetition time (TR), 2530 ms; with four echo times (TE1=1.66 ms; TE2= 3.53 ms; TE3= 5.40 ms; TE4= 7.27 ms); inversion time (TI), 1100 ms; flip angle, 7°; and 1mm isotropic resolution] was acquired. A diffusion q-ball sequence [repetition time (TR), 8000ms; echo time (TE), 119ms; 60 gradient directions obtained with b-value of 3000 s/mm^2 and 8 baseline b=0 images, 48 slices and isotropic resolution, 2.5mm] was acquired[6]. PET data were collected 0-90 min post-radiotracer injection. MR-based attenuation correction was applied using Statistical Parametric Mapping (SPM)–based, pseudo–computed tomography methodology[7]. PET data were binned (3600 s x 1, 300 s x 6) and reconstructed using the three-dimensional ordinary Poisson ordered-subset expectation maximization (3D OP-OSEM) algorithm[8,9] in units of SUV. The final PET data were reconstructed into 153 slices with 256 × 256 pixels and a 1.25-mm isotropic voxel size.

MR data analysis. In order to determine regions of interest in native space, the MEMPRAGE images were reconstructed with FreeSurfer's automated segmentation and parcellation, version 6.0 (http://surfer.nmr.mgh.harvard.edu/)[10]. Brain volumes for gray matter, white matter and cerebrospinal fluid as well as estimated total intracranial volume were obtained using Freesurfer. Diffusion MR (dMRI) images were preprocessed utilizing in house scripts, which include the following: axis alignment, centering, eddy current correction and, motion correction (https://github.com/pnlbwh/pnlutil). dMRI

images were visually quality checked for noise, severe motion, and signal drops. All images were masked to exclude non-brain areas with 3D Slicer[11]. To assess white matter microstructure, generalized fractional anisotropy (gFA) maps were calculated from the orientation distribution function (ODF) that were estimated at each voxel utilizing regularized spherical harmonics[12]. The gFA maps were resampled to 1-mm isotropic voxel size and registered to Montreal Neurological Institute (MNI) space in FSL [FMRIB (Oxford Centre for Functional MRI of the Brain) Software Library] using linear [FLIRT (FMRIB's linear image registration tool)][13,14] and nonlinear [FNIRT (FMRIB's nonlinear image registration tool)][15] algorithms. Whole brain voxel-wise analysis was performed with unsmoothed gFA images in MNI space using FSL's randomise[16].

Supplementary References

1. Scahill, R. I. *et al.* A Longitudinal Study of Brain Volume Changes in Normal Aging Using Serial Registered Magnetic Resonance Imaging. *Arch. Neurol.* **60**, 989 (2003).
2. Wey, H. Y. *et al.* Insights into neuroepigenetics through human histone deacetylase PET imaging. *Sci. Transl. Med.* **8**, (2016).
3. Catana, C. *et al.* Toward Implementing an MRI-Based PET Attenuation-Correction Method for Neurologic Studies on the MR-PET Brain Prototype. *J. Nucl. Med.* **51**, 1431–1438 (2010).
4. Tisdall, M. D. *et al.* Prospective motion correction with volumetric navigators (vNavs) reduces the bias and variance in brain morphometry induced by subject motion. *Neuroimage* **127**, 11–22 (2016).
5. Tisdall, M. D. *et al.* Volumetric navigators for prospective motion correction and selective reacquisition in neuroanatomical MRI. *Magn. Reson. Med.* **68**, 389–399 (2012).
6. Tuch, D. S. Q-ball imaging. *Magn. Reson. Med.* **52**, 1358–1372 (2004).
7. Izquierdo-Garcia, D. *et al.* An SPM8-Based Approach for Attenuation Correction Combining Segmentation and Nonrigid Template Formation: Application to Simultaneous PET/MR Brain Imaging. *J. Nucl. Med.* **55**, 1825–1830 (2014).

8. Chonde, D. B., Izquierdo-Garcia, D., Chen, K., Bowen, S. L. & Catana, C. Masamune: a tool for automatic dynamic PET data processing, image reconstruction and integrated PET/MRI data analysis. *EJNMMI Phys.* **1**, A57 (2014).

9. Conde, D. B. Improved PET Data Quantification in Simultaneous PET / MR Neuroimaging. (Harvard University, Graduate School of Arts & Sciences, 2015).

10. Fischl, B. *et al.* Whole brain segmentation: Automated labeling of neuroanatomical structures in the human brain. *Neuron* **33**, 341–355 (2002).

11. Norton, I. *et al.* SlicerDMRI: Open source diffusion MRI software for brain cancer research. *Cancer Res.* **77**, e101–e103 (2017).

12. Descoteaux, M., Angelino, E., Fitzgibbons, S. & Deriche, R. Regularized, fast, and robust analytical Q-ball imaging. *Magn. Reson. Med.* **58**, 497–510 (2007).

13. Jenkinson, M. & Smith, S. A global optimisation method for robust affine registration of brain images. *Med. Image Anal.* **5**, 143–156 (2001).

14. Jenkinson, M., Bannister, P., Brady, M. & Smith, S. Improved optimization for the robust and accurate linear registration and motion correction of brain images. *Neuroimage* **17**, 825–841 (2002).

15. Andersson, J. L. R., Jenkinson, M. & Smith, S. Non-linear registration, aka spatial normalisation. FMRIB Technial Report TR07JA2. *Oxford Cent. Funct. Magn. Reson. Imaging Brain, Dep. Clin. Neurol. Oxford Univ. Oxford, UK* 22 (2007).

16. Winkler, A. M., Ridgway, G. R., Webster, M. A., Smith, S. M. & Nichols, T. E. Permutation inference for the general linear model. *Neuroimage* **92**, 381–397 (2014).
