# Supplementary material for: Neuroepigenetic signatures of age and sex in the living human brain
Source: Nat Commun. 2019 Jul 3;10:2945. doi: 10.1038/s41467-019-11031-0 (PMC6610136; doi:10.1038/s41467-019-11031-0)
Supplement: Supplementary file 2 — Reporting Summary [file 41467_2019_11031_MOESM2_ESM.pdf]

nature research

Corresponding author(s): Jacob M. Hooker

Last updated by author(s): May 29, 2019

Reporting Summary

Nature Research wishes to improve the reproducibility of the work that we publish. This form provides structure for consistency and transparency in reporting. For further information on Nature Research policies, see Authors & Referees and the Editorial Policy Checklist.

Statistics

For all statistical analyses, confirm that the following items are present in the figure legend, table legend, main text, or Methods section.

n/a	Confirmed	
☐	☒	The exact sample size (*n*) for each experimental group/condition, given as a discrete number and unit of measurement
☐	☒	A statement on whether measurements were taken from distinct samples or whether the same sample was measured repeatedly
☐	☒	The statistical test(s) used AND whether they are one- or two-sided<br>*Only common tests should be described solely by name; describe more complex techniques in the Methods section.*
☐	☒	A description of all covariates tested
☐	☒	A description of any assumptions or corrections, such as tests of normality and adjustment for multiple comparisons
☐	☒	A full description of the statistical parameters including central tendency (e.g. means) or other basic estimates (e.g. regression coefficient) AND variation (e.g. standard deviation) or associated estimates of uncertainty (e.g. confidence intervals)
☐	☒	For null hypothesis testing, the test statistic (e.g. *F*, *t*, *r*) with confidence intervals, effect sizes, degrees of freedom and *P* value noted<br>*Give P values as exact values whenever suitable.*
☒	☐	For Bayesian analysis, information on the choice of priors and Markov chain Monte Carlo settings
☒	☐	For hierarchical and complex designs, identification of the appropriate level for tests and full reporting of outcomes
☐	☒	Estimates of effect sizes (e.g. Cohen's *d*, Pearson's *r*), indicating how they were calculated

Our web collection on statistics for biologists contains articles on many of the points above.

Software and code

Policy information about availability of computer code

Data collection	MR-PET data was collected simultaneously using a 3T Siemens TIM Trio scanner containing a BrainPET camera insert and an eight channel MR head coil. MR-based attenuation correction was applied using Statistical Parametric Mapping (SPM)–based, pseudo–computed tomography methodology and PET data were reconstructed using the three-dimensional ordinary Poisson ordered-subset expectation maximization (3D OP-OSEM) algorithm using custom codes developed at the Athinoula A. Martinos Center for Biomedical Imaging. Further information and custom codes are available upon reasonable request. Biochemical data were collected with a Chemidoc XRS imager using Image lab software version 5.2.1.
Data analysis	MR-PET image analysis was performed using a variety of analysis tools: FreeSurfer version 6.0 including automated parcellation and segmentation; PETSurfer; mri_coreg; 3D Slicer; FSL [FMRIB (Oxford Centre for Functional MRI of the Brain) Software Library] version 5.0.7 including MCFLIRT, randomise, and FEAT; FLIRT (FMRIB's linear image registration tool); FNIRT (FMRIB's nonlinear image registration tool); and PMOD version 3.4. Further information and custom codes are available upon reasonable request. Biochemical image analysis was performed with Image J.

For manuscripts utilizing custom algorithms or software that are central to the research but not yet described in published literature, software must be made available to editors/reviewers. We strongly encourage code deposition in a community repository (e.g. GitHub). See the Nature Research guidelines for submitting code & software for further information.

Data

Policy information about availability of data

All manuscripts must include a data availability statement. This statement should provide the following information, where applicable:

- Accession codes, unique identifiers, or web links for publicly available datasets
- A list of figures that have associated raw data
- A description of any restrictions on data availability

The data that support these findings are available from the corresponding author, J.M.H., upon reasonable request. Human subject data will be deidentified to protect confidentiality.

Custom codes for MR and PET data processing are available from the corresponding author, J.M.H., upon reasonable request.

Field-specific reporting

Please select the one below that is the best fit for your research. If you are not sure, read the appropriate sections before making your selection.

☒ Life sciences ☐ Behavioural & social sciences ☐ Ecological, evolutionary & environmental sciences

For a reference copy of the document with all sections, see nature.com/documents/nr-reporting-summary-flat.pdf

Life sciences study design

All studies must disclose on these points even when the disclosure is negative.

Sample size	Forty-two eligible subjects, twenty females and twenty-two males, underwent MR-PET scanning. All subject data collected at the time of manuscript preparation were included. Based on previously published PET imaging studies on age and sex, the sample size in this study is appropriate.
Data exclusions	Forty-two subjects underwent an MR-PET scan, but one male subject was excluded from primary analyses for a dental work-related artifact. Exclusion occurred as a result of failing a quality control check of the reconstructed PET data.
Replication	To verify the reproducibility of the main experimental findings, two independent normalization strategies were applied using 1) SUV normalized to whole brain mean and 2) SUV normalized to pons. The results were consistent between both methods.
Randomization	This is not relevant to this study as all subjects were injected with the PET radiotracer.
Blinding	Investigators were not blinded during data collection or analysis. Blinding was not relevant to this imaging study because age and sex variables were input into statistical models after all the data sets were simultaneously processed. Blinding was not performed during the biochemical study to ensure that old and young donors would be intermixed during western blotting, to alleviate potential location artifacts. No outliers were excluded during imaging or biochemical analysis.

Reporting for specific materials, systems and methods

We require information from authors about some types of materials, experimental systems and methods used in many studies. Here, indicate whether each material, system or method listed is relevant to your study. If you are not sure if a list item applies to your research, read the appropriate section before selecting a response.

Materials & experimental systems

n/a	Involved in the study	
☐	☒	Antibodies
☒	☐	Eukaryotic cell lines
☒	☐	Palaeontology
☒	☐	Animals and other organisms
☐	☒	Human research participants
☒	☐	Clinical data

Methods

n/a	Involved in the study	
☒	☐	ChIP-seq
☒	☐	Flow cytometry
☐	☒	MRI-based neuroimaging

Antibodies

Antibodies used	Primary:<br>HDAC1: Thermo Fisher (supplier), Invitrogen #PA1-860 (catalog #) at 1:6000, polyclonal, lot #SD247979<br>HDAC2: Abcam (supplier) #ab124974 (catalog #) at 1:5000, rabbit monoclonal clone EPR5001, lot #GR97402-7<br>HDAC3: Abcam (supplier) #ab32369 (catalog #) at 1:2500,rabbit monoclonal clone Y415, lot #'s GR320398-7,-8, GR3173923-1<br>HDAC6: Santa Cruz (supplier, no longer available) #sc11420 (H-300) (catalog #) at 1:6000, rabbit monoclonal IgG, lot # G1515<br>GAPDH: Abcam (supplier),#ab8245 (catalog #) at 1:2000000, mouse monoclonal clone 6C5, lot#'s GR232949-5, GR3207992-1<br>Secondary:<br>anti-rabbit-HRP: Cell signaling (supplier), #7074S (catalog #) at 1:5000, species of origin: goat, lot #'s 27,28<br>anti-mouse HRP: Cell Signaling (supplier), #7076S (catalog #) at 1:5000, species of origin: goat, lot #32
Validation	All antibodies used in this current have been published in: H.-Y. Wey, T. M. Gilbert, N. R. Zürcher, A. She, A. Bhanot, B. D. Taillon, F. A. Schroeder, C. Wang, S. J. Haggarty, J. M. Hooker, Insights into neuroepigenetics through human histone deacetylase PET imaging. Sci. Transl. Med. 8, 351ra106 (2016). In house quality control reported in this publication (e.g. recombinant verification, off-target assessment)<br><br>HDAC1: According to supplier, reacts with dog, hamster, human, mouse, rat, tested in Western blot and other applications with 36 published figures and 56 references, RRID:AB_2118091

HDAC2: According to supplier, reacts with mouse, rat, human, tested in Western blot and other applications, RRID:AB_11127303.
HDAC3: According to supplier, reacts with mouse, rat, human, tested in Western blot and other applications with a number of references, RRID:AB_732780 .
HDAC6: According to supplier, recommended for detection of HDAC6 of human origin by Western blot and other applications, RRID:AB_2116634.
GAPDH: According to supplier, reacts with mouse, rat, rabbit, human and other species, tested in Western blot and other applications, RRID:AB_2107448.
http://antibodyregistry.org [Research Resource Identifiers (RRIDs)]

Human research participants

Policy information about studies involving human research participants

Population characteristics	Relevant covariates include age and sex of human research participants.
Recruitment	Healthy volunteers were recruited through advertising with flyers in the general Boston community, web announcements and Partners Clinical Trials Research Portal.
Ethics oversight	The study was approved by the Partners HealthCare Institutional Review Board (IRB) and the Massachusetts General Hospital (MGH) Radioactive Drug Research Committee. All subjects provided written informed consent according to the Declaration of Helsinki.

Note that full information on the approval of the study protocol must also be provided in the manuscript.

Magnetic resonance imaging

Experimental design

Design type	structural and diffusion only
Design specifications	structural and diffusion scans
Behavioral performance measures	NA

Acquisition

Imaging type(s)	structural
Field strength	3T
Sequence & imaging parameters	A T1-weighted multi-echo MPRAGE sequence (MEMPRAGE) with epi navigator [repetition time (TR), 2530 ms; with four echo times (TE1=1.66 ms; TE2= 3.53 ms; TE3= 5.40 ms; TE4= 7.27 ms); inversion time (TI), 1100 ms; flip angle, 7°; and 1mm isotropic resolution] was acquired.
Area of acquisition	whole brain

Diffusion MRI ☒ Used ☐ Not used

Parameters: A diffusion q-ball sequence [repetition time (TR), 8000ms; echo time (TE), 119ms; 60 gradient directions obtained with b-value of 3000 s/mm2 and 8 baseline b=0 images, 48 slices and isotropic resolution, 2.5mm] was acquired.

Preprocessing

Preprocessing software	Diffusion MR (dMRI) images were preprocessed utilizing in house scripts, which include the following: axis alignment, centering, eddy current correction and, motion correction (https://github.com/pnlbwh/pnlutil).
Normalization	The gFA maps were resampled to 1-mm isotropic voxel size and registered to Montreal Neurological Institute (MNI) space in FSL [FMRIB (Oxford Centre for Functional MRI of the Brain) Software Library] using linear [FLIRT (FMRIB's linear image registration tool)] and nonlinear [FNIRT (FMRIB's nonlinear image registration tool)] algorithms.
Normalization template	MNI152
Noise and artifact removal	dMRI images were visually quality checked for noise, severe motion, and signal drops. All images were masked to exclude non-brain areas with 3D Slicer.
Volume censoring	NA

Statistical modeling & inference

Model type and settings	Whole brain voxel-wise analysis was performed with unsmoothed gFA images in MNI space using FSL's randomise.

Effect(s) tested: gFA association with age

Specify type of analysis: ☒ Whole brain ☐ ROI-based ☐ Both

Statistic type for inference (See Eklund et al. 2016): voxel-wise, non-parametric with FSL randomise

Correction: Family wise error correction

Models & analysis

n/a	Involved in the study
☒	☐ Functional and/or effective connectivity
☒	☐ Graph analysis
☒	☐ Multivariate modeling or predictive analysis
